# METANet: a supervised ensemble learning framework for reconstructing direct and functional tissue-specific transcription factor networks

**DOI:** 10.1093/bioinformatics/btag738

**Published:** 2026-10-07

**Authors:** Wooseok J Jung, Sandeep Acharya, Daniel P Ruskin, Shu Liao, Vaha Akbary Moghaddam, Zolboo Erdenebaatar, Michael R Brent

**Affiliations:** Department of Computer Science and Engineering, Washington University, St. Louis, MO 63130, United States; Center for Genome Sciences and Systems Biology, Washington University School of Medicine, St. Louis, MO 63110, United States; Center for Genome Sciences and Systems Biology, Washington University School of Medicine, St. Louis, MO 63110, United States; Division of Computational and Data Sciences, Washington University, St. Louis, MO 63130, United States; College of Information, University of Maryland, College Park, MD 20742, United States; Department of Computer Science and Engineering, Washington University, St. Louis, MO 63130, United States; Center for Genome Sciences and Systems Biology, Washington University School of Medicine, St. Louis, MO 63110, United States; Division of Statistical Genomics, Washington University School of Medicine, St. Louis, MO 63110, United States; Department of Computer Science and Engineering, Washington University, St. Louis, MO 63130, United States; Center for Genome Sciences and Systems Biology, Washington University School of Medicine, St. Louis, MO 63110, United States; Department of Computer Science and Engineering, Washington University, St. Louis, MO 63130, United States; Center for Genome Sciences and Systems Biology, Washington University School of Medicine, St. Louis, MO 63110, United States; Department of Genetics, Washington University School of Medicine, St. Louis, MO 63110, United States

## Abstract

**Motivation:**

Reconstructing tissue-specific transcription factor (TF) networks remains challenging. TF-motif-based methods often lack functional validation, while expression-based methods struggle to distinguish direct binding from indirect regulation. Integration of diverse data types is necessary to accurately prioritize likely functional targets directly bound by TFs across human tissues.

**Results:**

We introduce METANet (Motif Expression TF Association Networks), a supervised ensemble learning framework that combines TF motifs, cis-regulatory element activity, and linear and nonlinear expression-derived features to predict TF binding. Applied to 36 human tissues, predicted METANet maps significantly outperform established methods in identifying direct, likely functional targets of TFs validated by ChIP-seq and gene ontology. Furthermore, METANet maps capture tissue-specific regulation comparable to existing methods, allowing the identification of reproducible gene–trait associations.

**Availability and implementation:**

All code and network maps are freely available at Zenodo https://doi.org/10.5281/zenodo.20614479.

## 1 Introduction

A transcription factor (TF) network map is a directed graph in which nodes represent TFs and genes, and edges link TFs to the genes they directly regulate. Numerous algorithms have been developed for mapping TF networks from a variety of data types, and these methods generally fall into two broad categories. The first category uses TF binding evidence, such as DNA sequence motifs (Janky *et al.* 2014) or TF ChIP-seq (Gerstein *et al.* 2012), often combined with promoter and enhancer annotations to define regulatory elements. Motif data describe locations where a TF could bind, and TF ChIP-seq data reveal the genomic regions where a TF actually binds. However, binding evidence alone cannot confirm whether a TF functionally affects the transcription rates of its target genes (TGs). For instance, many TF-bound motifs have no apparent effect on gene transcription (White *et al.* 2013, Cusanovich *et al.* 2014, Slattery *et al.* 2014). Furthermore, most motifs for a TF are not bound by the TF (Wang *et al.* 2012, Dror *et al.* 2015), and many sites where a TF binds lack motifs (Worsley Hunt and Wasserman 2014, Horton *et al.* 2023).

Another class of methods uses gene expression measurements under baseline conditions or after perturbations of TF expression (Langfelder and Horvath 2008, Huynh-Thu *et al.* 2010, Haynes *et al.* 2013, Skok Gibbs *et al.* 2022, Abid and Brent 2023, Kang *et al.* 2018). While perturbation-response data can reveal the functional effects of TFs, they do not confirm direct binding. Moreover, expression-based methods are prone to capturing indirect effects, as correlations between TF and TG expression may arise from effects such as shared upstream regulators or mediated interactions (Feizi *et al.* 2013, Mercatelli *et al.* 2020, Xiao *et al.* 2022). To address these limitations, prior studies have established that integrating sequence-specific motifs as priors with gene expression data significantly improves regulatory network inference compared to expression-based inference alone (Greenfield *et al.* 2013, Roy *et al.* 2013, Siahpirani and Roy 2017).

While many regulatory relationships are shared across tissues, some TF–TG interactions are context specific. TF network maps inferred from gene expression and binding data have been shown to vary significantly across cell types and tissues (Melé *et al.* 2015, Sonawane *et al*. 2017b, Gamazon *et al.* 2018, Haigis *et al.* 2019). Our group has previously mapped TF networks in *Saccharomyces cerevisiae* and *Drosophila melanogaster* using both TF binding and gene expression data, successfully capturing direct and functional targets of TFs (Kang *et al.* 2018, Abid and Brent 2023). However, these mapping efforts focused on a single, aggregate context and did not address regulatory variation across tissues, which limits their utility for understanding context-specific gene regulation in human tissues. Most widely used approaches for tissue-specific TF network mapping remain limited in their ability to capture direct and functional targets. For example, TF motif-based approaches using cis-regulatory element (CRE) activity levels across tissues can suggest tissue-specific binding potential, but do not confirm functional relationships (Marbach *et al.* 2016), while correlation-based networks fail to account for the physical binding of the TF in the TG’s CREs (Pierson *et al.* 2015, Saha *et al.* 2017). One notable exception is (Sonawane *et al*. 2017b), which uses PANDA, combining TF motifs and protein–protein interactions (PPI) with tissue-specific expression data in a message-passing framework, to map 38 tissue-specific TF networks. Building on our prior work, we introduce the METANet (Motif Expression TF Association Network) framework, a supervised machine learning approach to predict tissue-specific direct and likely functional TF–TG regulatory relationships. Unlike PANDA’s unsupervised approach, METANet uses supervised learning, which allows the model to learn directly from TF ChIP-seq-based binding signals. Using tissue-specific gene expression data and TF network maps derived from Marbach *et al.* (2016) as predictor variables, METANet predicts the probability that a TF binds a TG in TF-ChIP-seq data the model has not seen.

This paper makes four contributions. We provide a comprehensive resource of human tissue-specific TF network maps across 36 tissues, encompassing ∼237 TFs and ∼12 150 protein-coding genes. We evaluate the ability of our TF network maps to capture direct and likely functional targets of TFs using four evaluation metrics and compare our performance to the networks proposed by Sonawane *et al.* We also compare to the original networks from Marbach *et al.* (which does not use gene expression data). Across 36 tissues, we show that predicted METANet maps better capture both the putative direct and functional targets of TFs compared to benchmark network maps. We also found that the tissue specificity of predicted METANet maps is comparable to that of the network maps from Marbach *et al.* and Sonawane *et al.* To explore potential applications of the predicted METANet maps, we input them into FISHNET (Acharya *et al.* 2025), a network-driven gene prioritization method, using transcriptome-wide association (TWAS) summary statistics for traits associated with cardiovascular risk from the Long Life Family Study (LLFS) cohort. FISHNET using the predicted METANet map identified replicated gene–trait associations that were missed when other networks were passed as inputs. Furthermore, the predicted METANet map identified regulatory mechanisms that may mediate the effects of known trait-associated variants.

## 2 Methods

### 2.1 Motif expression TF association networks

The METANet framework combines five distinct sets of regulatory edge features using XGBoost: a tissue-specific motif-based feature (Marbach) and four expression-based features derived by applying Least Absolute Shrinkage and Selection Operator (LASSO) and Bayesian Additive Regression Trees (BART) (Fig. 1a). The Marbach scores are based on motif occurrences weighted by tissue-specific CRE activity. LASSO and BART scores are derived from regressing each gene’s mRNA levels on those of all TFs using either tissue-specific samples or samples aggregated across all tissues. These five weighted networks form a set of five features that are trained with binary labels extracted from binding experiments: 1 when there is evidence of direct binding within the CREs of a gene and 0 otherwise.

**Figure 1:**
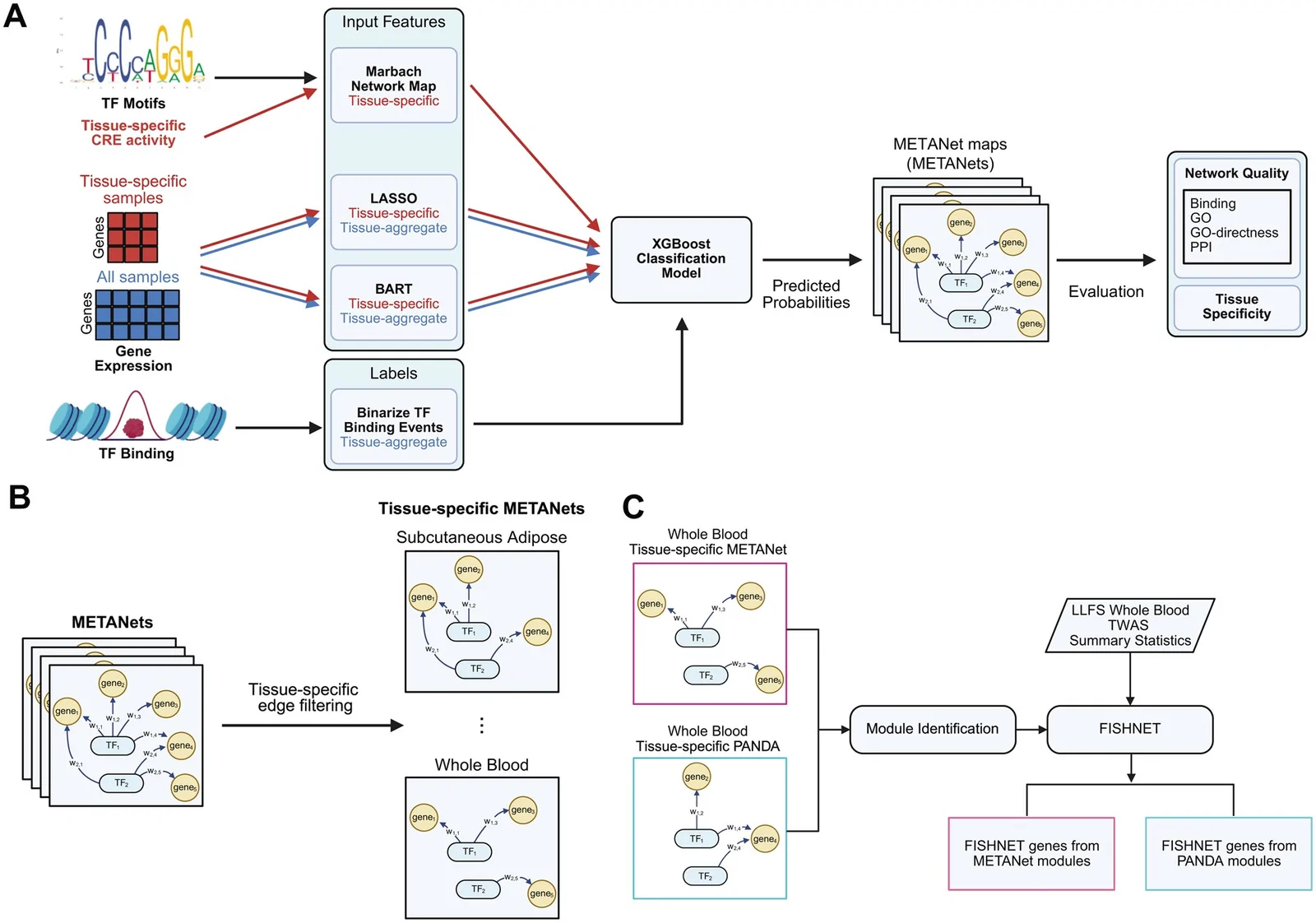
METANet framework overview. (a) For each tissue, the METANet framework uses XGBoost to combine evidence scores from tissue-specific TF network maps from Marbach *et al.* (2016), tissue-specific and tissue-aggregate LASSO and BART regression models from GTEx expression data, and binarized TF binding labels. The predicted probabilities from the XGBoost model are used as edge scores in our TF network maps. Red arrows indicate tissue-specific data, while blue arrows indicate tissue-aggregate data. The predicted METANet maps (METANets) are evaluated for network quality and tissue specificity. (b) Tissue-specific METANet maps are inferred from the predicted METANet maps by filtering for tissue-specific edges. (c) Modules identified from the whole-blood-specific METANet map and PANDA network are used in FISHNET with TWAS summary statistics from the LLFS to identify FISHNET genes.

We trained the model using 10-fold nested cross-validation (CV) with TF-disjoint partitioning to ensure that the model is evaluated on its ability to generalize to unseen TFs. This prevents the model from leveraging TF-specific patterns learned during training when making predictions for test fold edges. Hyperparameter tuning was performed using three-fold inner CV, also with TF-disjoint partitioning, ensuring that no TF leakage occurred at either level of the nested procedure. Bayesian optimization using the Tree-structured Parzen Estimator algorithm (Bergstra *et al.* 2011) was used to maximize the area under the precision-recall curve (AUPRC) on the inner CV folds. Early stopping applied during model fitting using log-loss as the internal evaluation metric to determine the optimal number of boosting rounds. The optimal hyperparameters were used to train a final XGBoost model on all training data to generate predictions for the held-out test fold. A TF was not allowed to regulate itself.

### 2.2 Construction of binding labels

For each TF in our network, defined according to the curated catalog of human TFs from Lambert *et al.* (2018), we downloaded all available individual ChIP-seq experiment files from REMAP 2020 (Chèneby *et al.* 2020) and retained only high-confidence peaks (FDR *q*-value ≤ 0.01). Peaks across all experiments for a given TF were concatenated into a single set. For each nucleotide within a FANTOM5 promoter or enhancer region (Andersson *et al.* 2014, Forrest *et al*. 2014), a score was calculated as the maximum $-\log_{10}(\text{q-value})$ across all peaks overlapping that nucleotide. Gene-level scores were then calculated by summing nucleotide scores across all CREs associated with a TG (see Fig. S1 and Supplementary Methods, available as supplementary data at *Bioinformatics* online, for details).

Binding labels representing TF–gene binding events were constructed as follows. For each TF, genes with a non-zero binding score were considered candidate positives. If the number of candidate positives exceeded 10% of the gene universe, only the top 10% of highest-scoring genes were retained as positive labels; otherwise, all genes with non-zero binding scores were assigned positive labels. As a result, the 10% threshold acts as a ceiling rather than a fixed target, and the actual positive label rate is ∼8.7% , averaged across tissues. All remaining TF–gene pairs were labeled as negative. While some genuine binding events may be excluded by the significance threshold, that would be true at any threshold, and labeling these as negatives is standard in the field. The robustness of our results to alternative label definitions (5% and 20% thresholds) is assessed in Figs S2–S5, available as supplementary data at *Bioinformatics* online.

### 2.3 Construction of expression-based features

We downloaded bulk RNA-seq read counts and transcripts per million (TPM) data from the Genotype-Tissue Expression (GTEx) consortium V8 release (The GTEx Consortium 2020). The GTEx read count data for protein-coding genes were filtered to remove lowly expressed genes. Specifically, genes with ≤3 counts per million in ≥98.5% of samples were removed.

We constructed the gene expression-based features by training two regression algorithms, LASSO and BART, to predict the expression level of each gene from the expression levels of all TFs (Chipman *et al.* 2010, Haynes *et al.* 2013, Kang *et al.* 2018, Abid and Brent 2023). LASSO is a linear regression method with L1 regularization. BART is a nonparametric sum-of-trees model that captures complex, nonlinear relationships through an ensemble of regression trees. Features for a given TF–gene pair reflect the effect of the TF’s expression level on the gene’s predicted expression level in the trained models. For example, the feature extracted from a LASSO model is the learned coefficient of the TF’s expression level. Both algorithms are run on the filtered tissue-specific gene expression TPM data and, separately, on the aggregated TPM data from all GTEx tissue samples, yielding 4 regression features for each TF–gene pair.

### 2.4 Marbach networks

We downloaded 394 cell-type- and tissue-specific networks from Marbach *et al.* (2016). We formed 36 tissue networks, merging closely related networks by taking the graph union (union of the node and edge sets while retaining the maximum weight for each edge). To guide this merging process, we adopted the tissue-to-cell mapping established by Marbach *et al.* for 13 GTEx tissues. We then extended this by manually curating mappings for the remaining 23 tissues using annotations from the BRENDA tissue ontology (Gremse *et al.* 2011) (see Table S1, available as supplementary data at *Bioinformatics* online).

### 2.5 PANDA networks

For benchmarking, we obtained PANDA networks for the same 26 tissues from Sonawane *et al*. (2017a, 2017b). We processed both fully connected and tissue-specific PANDA networks identically to the METANet maps, restricting analyses to protein-coding genes and the common set of TFs to ensure fair comparison (see Supplementary Methods, available as supplementary data at *Bioinformatics* online).

### 2.6 Network quality evaluation

#### 2.6.1 Binding metric

We calculated the percentage of top-scoring TF–TG edges supported by TF binding data at different thresholds. Top-scoring edges were defined by sorting all edges by the edge score in descending order. Thresholds were scaled to the number of TGs per TF. This is an average, so different TFs have different numbers of TGs at each threshold. A higher percentage of binding support reflects greater enrichment of direct edges in the network map. The random expectation is the probability that a randomly selected TF–TG edge is supported by the binding data.

#### 2.6.2 Gene ontology metric

We performed Gene Ontology (GO) enrichment analysis for each TF’s targets using GO-Term-Finder v0.86 (Boyle *et al.* 2004). To focus on terms associated with specific biological processes, rather than extremely generic terms such as ‘biosynthetic process’, we excluded terms with number of annotated genes below 5 or exceeding 300. Each TF’s score was the maximum –log *P*-value over all GO terms. If a TF’s target set showed no significant enrichment for any GO biological process, we assigned the TF a *P*-value of 1 (equivalent to –log *P* = 0). The final network-level score was taken as the median of these scores across all TFs. We did these analyses for each threshold, as in the binding metric, but without excluding TFs that did not have binding data available.

#### 2.6.3 GO-directness metric

To determine whether improvements on the GO evaluation metric came at the cost of including indirect edges, we calculated, for each TF, the percentages of binding support for edges that matched the GO term with the highest -log P-value. For each threshold, we reported the average percentage of binding support across TFs. The random expectation is identical to that of the Binding metric.

#### 2.6.4 PPI metric

First, we calculated the Jaccard similarity between the sets of TGs of each (TF, TF) pair; the Jaccard similarity is 1 when the target sets are the same and 0 when none of the targets in the sets are shared. Next, we sorted (TF, TF) pairs by Jaccard similarity and calculated the percentage of the top 50 and 100 pairs that are supported by physical interactions from the PPI STRING database (Szklarczyk *et al.* 2023). The database has 477 903 interactions predicted with high confidence (STRING score ≥ 0.7). A higher percentage of PPI support means that (TF, TF) pairs are likely to work as a part of a known physical protein complex. We did these analyses for the TF–TG edges at threshold of 250 targets per TF. The random expectation is the probability that a randomly selected pair of TFs present in a tissue network is supported by the PPI database. This probability was computed separately for each tissue and then averaged across the 36 tissues.

#### 2.6.5 Empirical null

To establish an empirical null to evaluate network quality, we generated 50 permuted networks by randomly shuffling the fully connected METANet edge scores (see Fig. S6, available as supplementary data at *Bioinformatics* online). Each permuted network was subjected to the same cutoff thresholds as the real networks and evaluated across all four metrics, providing a consistent null distribution. Differences between network maps on each evaluation metric were assessed with paired *t*-tests across tissues, and *P*-values were adjusted for multiple testing using the Benjamini–Hochberg procedure within each test family, defined as the set of comparisons for one evaluation metric within one network comparison group (i.e. one figure panel).

### 2.7 Tissue-specificity evaluation

#### 2.7.1 Tissue-specific edge filtering

Tissue-specific regulatory edges were identified for METANets and PANDA networks using the interquartile range (IQR)-based filtering specificity score described by Sonawane *et al*. (2017b). Edges with a normalized specificity score >2 were retained as tissue-specific (see Supplementary Methods, available as supplementary data at *Bioinformatics* online). The number of TFs, genes, and edges in each tissue-specific METANet and PANDA network is reported in Table S2 and Fig. S7, available as supplementary data at *Bioinformatics* online.

#### 2.7.2 Validation of tissue specificity using eQTL data

For each tissue, we collected all eQTLs that are active in that tissue from GTEx. Using these eQTLs, we ranked the networks constructed for each tissue. Within each network, an edge was considered supported if an eQTL modified the expression level of the TG and occurred in a CRE for the TG that also contained a binding peak for the TF (The GTEx Consortium 2020). We calculated ‘eQTL support’ and ‘eQTL count’ metrics (see Supplementary Methods, available as supplementary data at *Bioinformatics* online). For each tissue significance against the null hypothesis of no tissue correspondence was assessed using a permutation test (10 000 iterations) on the sum of ranks for matching tissue pairs (Fig. S6, available as supplementary data at *Bioinformatics* online). Permutation testing is appropriate here because the null distribution of the rank-sum statistic is not analytically tractable under the tissue-pairing design, making a data-driven null the natural choice.

Networks were compared against each other using the Wilcoxon signed-rank test on the distributions of matching tissue ranks across tissues. The Wilcoxon signed-rank test was chosen over a paired *t*-test for this comparison because the matching tissue ranks derived from the IQR-based filtering procedure may not be normally distributed, and the nonparametric test makes no distributional assumption about the pairwise differences.

#### 2.7.3 Evaluation against cell line-specific binding labels

Because METANets were trained on tissue-aggregated binding labels (Section 2.2), we tested whether aggregation biases the evaluation by comparing against binding labels from individual cell lines. We selected three cell lines that correspond to tissues in our panel and are among the most extensively assayed in REMAP 2020: K-562 (whole blood), Hep-G2 (liver), and MCF-7 (breast mammary tissue). For each cell line, labels were built from that cell line’s REMAP 2020 experiments alone, using the per-TF top 10% procedure of Section 2.2 on the same TF-gene pairs as the aggregate labels. All four label sets (aggregate and three cell lines) therefore share the same construction. Because the 10% threshold is a ceiling, positive rates still differ between label sets (∼8.9% for aggregate vs. ∼6.6% for MCF-7; Fig. S10, available as supplementary data at *Bioinformatics* online).

We evaluated the METANet and PANDA networks for each of the three tissues against all four label sets. For each TF with at least one positive label in a given label set, we computed per-TF AUROC and AUPRC. AUROC is the primary metric. AUPRC depends on the positive rate and is therefore reported for completeness (Fig. S10, available as supplementary data at *Bioinformatics* online) but not compared across label sets.

We asked two questions. First, to test whether METANets outperform PANDA regardless of the label set, we compared per-TF area under the receiver operator curve (AUROC) between the two methods for each tissue and label set using a paired Wilcoxon signed-rank test over TFs scored by both. Second, to test whether each tissue network performs best on its tissue-matched cell line, we compared per-TF AUROC on matched versus mismatched cell-line labels within each tissue using a paired Wilcoxon signed-rank test. To test for a matching effect across all three tissues together, we combined all per-TF AUROC values from tissue-matched network–cell-line pairs into one group and all values from mismatched pairs into a second group, and compared the two groups with a one-sided Mann-Whitney U test (matched > mismatched). We used an unpaired test because each tissue has one matched and two mismatched cell lines, so the two groups cannot be paired one-to-one. P-values for the METANet-versus-PANDA comparisons and for the per-tissue matched-versus-mismatched comparisons were corrected using the Benjamini-Hochberg procedure within each test family. The combined matched-versus-mismatched comparison was a single planned test and was not corrected.

### 2.8 Gene–trait association analysis using FISHNET

We evaluated the biological relevance of predicted METANets using FISHNET, a network-based gene prioritization tool (Acharya *et al.* 2025). FISHNET is designed to prioritize genes that exhibit suggestive association signals but are more likely than expected by chance to replicate across independent datasets. It operates on the intuition that genes with low *P*-values purely by chance are distributed randomly, whereas true associations tend to cluster within densely connected network modules and share common biological functions. Using the whole blood tissue-specific METANet and PANDA network as inputs, we partitioned networks into modules using the M1 modularity optimization algorithm (Tomasoni *et al.* 2020). We integrated TWAS *P*-values for 11 cardiovascular traits from the LLFS and assessed replication using summary statistics from the Framingham Heart Study (FHS).

## 3 Results

### 3.1 Overview of TF network map reconstruction

To map tissue-specific TF regulatory networks, we developed the METANet framework, a supervised learning approach that integrates TF motif data, gene-expression-derived features, and TF binding evidence. For each of 36 human tissues, we trained an XGBoost model (Chen and Guestrin 2016) to predict whether a TF binds within the CREs of a gene, using ChIP-seq data from REMAP 2020 (Chèneby *et al.* 2020) as ground truth (Fig. 1). Each instance in the model corresponds to a candidate TF–TG pair and was represented by five biologically motivated features: (1) a motif-based regulatory score from the tissue-specific Marbach network (Marbach *et al.* 2016), which combines TF motif occurrences with tissue-specific CRE activity; (2–5) four expression-derived features capturing both linear and nonlinear TF–TG relationships. The features were derived by regressing the expression of each gene on the expression of all TFs using both LASSO (Tibshirani 1996) and BART (Chipman *et al.* 2010). We extracted from the model, for each gene, measures of the relatedness of each TF’s expression to the gene’s expression (see Section 2 for details). Both regression algorithms were applied to both tissue-specific and tissue-aggregated RNA-seq data from GTEx (The GTEx Consortium 2020) (Fig. 1).

The binding labels used for model training are derived from tissue-aggregated ChIP-seq data, as tissue-resolved TF binding data at the scale required for genome-wide modeling across 36 tissues is not currently available. However, we hypothesized that the resulting predictions would nevertheless capture tissue-specific regulatory relationships, because the input features include tissue-specific gene expression data and tissue-specific CRE activity scores. The tissue specificity of predicted METANets is assessed empirically in Section 3.6.

We used 10-fold nested CV to train and evaluate each XGBoost model. Predicted probabilities from test folds were used as edge scores in the resulting predicted METANet maps (METANets), linking TFs to their likely direct and biologically coherent targets. In total, we mapped the TF networks of 36 human tissues, encompassing an average of 237 TFs and 12 150 genes. Classification performance metrics, hyperparameter values, and robustness evaluations across alternative binding thresholds are provided in Tables S3–S5 and Figs S2–S5, available as supplementary data at *Bioinformatics* online. Across tissues, the model achieves a mean test sensitivity of 0.456 ± 0.024 and mean test specificity of 0.676 ± 0.022. These metrics are computed at the default 0.5 decision threshold under a class imbalance of approximately 10:1 negative-to-positive TF–gene edges, which tends to inflate specificity relative to sensitivity. The consistency between training, validation, and test performance indicates stable CV without overfitting. Additionally, the discriminative performance of METANet is robust to the binding label threshold across all 36 tissues (Fig. S8, available as supplementary data at *Bioinformatics* online). As the primary use of METANets is ranked prioritization of TF–gene interactions rather than binary classification, we direct the reader to the biological evaluation metrics in Sections 3.2–3.5 as the primary measure of model utility.

### 3.2 METANets capture direct and likely functional targets of TFs

We compared METANets against the motif-based Marbach networks, constructed using motif occurrences and tissue-specific CRE activity levels (Marbach *et al.* 2016), and PANDA networks from Sonawane *et al.* built using motifs and expression via an unsupervised approach (Sonawane *et al*. 2017b), across 26 common tissues. For the PANDA networks, we used the fully connected PANDA output networks from Sonawane *et al.* prior to filtering for tissue-specific edges. All networks we evaluated include continuous edge scores, from which discrete networks can be created by thresholding and retaining only edges above the chosen cutoff. To enable fair comparisons across tissues, we applied thresholds scaled to the average number of targets per TF (e.g. top 20 000 edges for 200 TFs implies 100 targets per TF), and we examined how evaluation results changed as more, lower-scoring edges were included.

To assess network quality, we used four complementary metrics: binding, GO, GO-directness, and PPI. To provide an empirical null expectation for each metric, we generated 50 random networks by permuting the edge scores of METANets. These random networks were also subjected to the same cutoff thresholds as other networks before evaluation.

#### 3.2.1 Binding

The Binding metric is the fraction of edges supported by binding data showing that the TF binds in one or more of the target’s CREs, sourced from the FANTOM5 database (Andersson *et al.* 2014, Forrest *et al*. 2014). METANets significantly exceeded both Marbach and PANDA (paired *t*-tests, *P* < .001 for each; Fig. 2a) and far exceeded the random expectation (*t*-test, $P<10^{-30}$), indicating stronger enrichment for directly bound targets. Paired *t*-tests were used throughout the Results (Sections 3.2–3.5) to compare network quality metrics across methods, with each pair of observations corresponding to the same tissue evaluated under both methods (*n* = 36 tissues for comparisons including Marbach; *n* = 26 tissues for comparisons including PANDA). Pairing by tissue is appropriate because tissues differ substantially in the number of expressed genes and TFs, and pairing removes this shared variance to increase statistical power for detecting method-level differences. The paired *t*-test does not require the individual tissue-level differences to be normally distributed; it requires that the sampling distribution of the mean difference be approximately normal, which is supported by the central limit theorem at our sample sizes (*n* = 36 and *n* = 26 tissues). *P*-values for these comparisons were adjusted for multiple testing using the Benjamini–Hochberg procedure within each test family as defined in Section 2.6.5. All *P*-values reported in Sections 3.2–3.5 and all significance annotations in Figs 2 and 3 reflect adjusted values.

**Figure 2:**
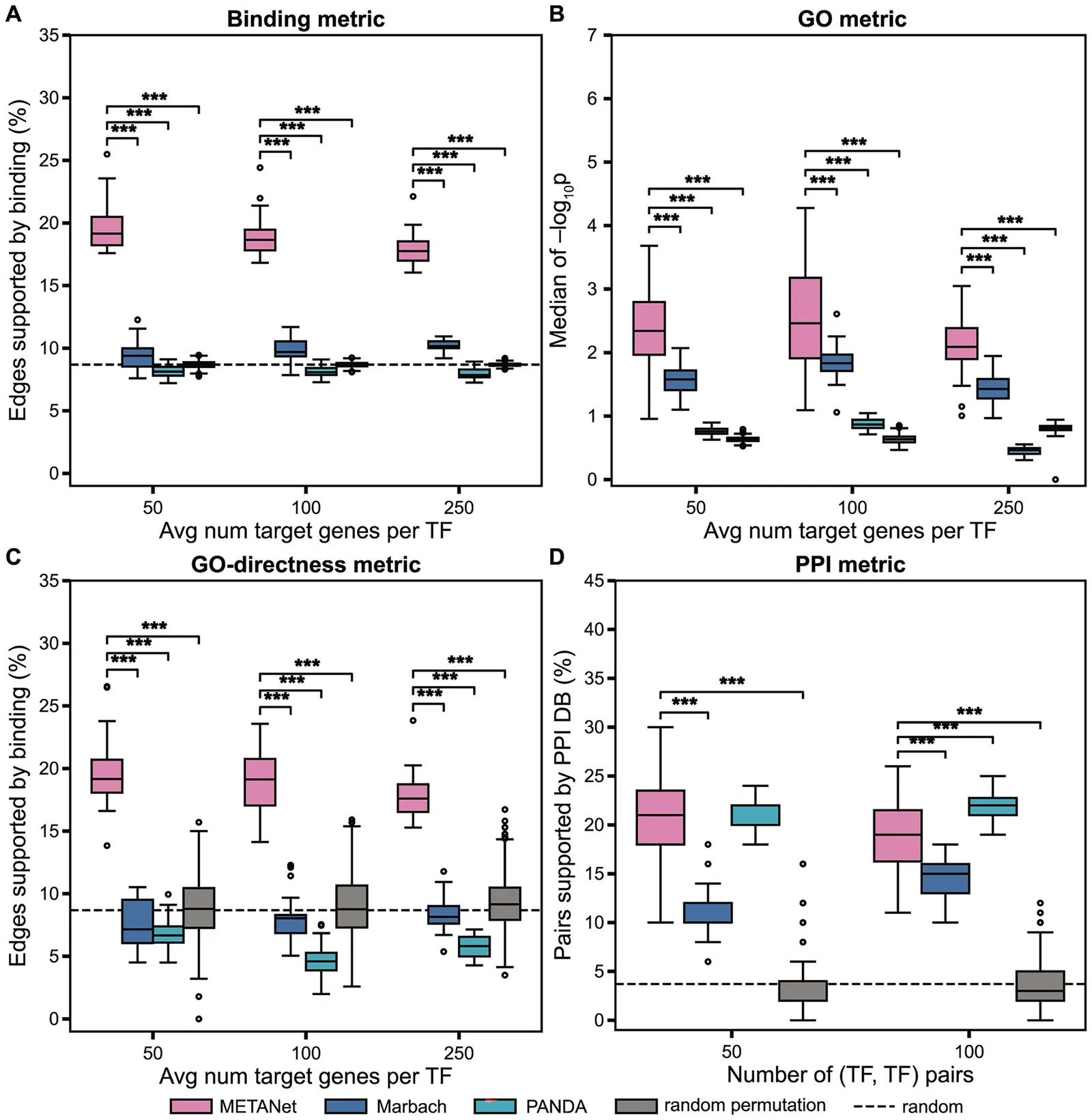
Evaluation of network quality. Performance of METANets, Marbach networks, and PANDA networks evaluated on 26 tissues for which all three networks were available. Each boxplot shows the distribution of metric values across the 26 tissues. Pink: METANets; blue: Marbach network maps; cyan: PANDA network maps; gray: random expectation based on permutations of METANets; black dashed: random expectation. Significance bars are drawn only for significant paired *t*-tests with METANets. *P ≤ .05, **P ≤ .01, ***P ≤ .001. Significance levels reflect Benjamini–Hochberg-adjusted *P*-values (see Section 2.6.5). Metric definitions are provided in Section 2.6. (a) Binding metric. Fraction of top-scoring TF–gene edges supported by TF ChIP-seq binding evidence within the cis-regulatory elements of the target gene. Random expectation is the fraction of all TF–gene edges supported by binding data. METANets significantly outperform both Marbach and PANDA networks at all thresholds. (b) GO metric. Median across TFs of the maximum − log_10_  *P*-value from GO biological process over-representation analysis of each TF’s predicted target set. The gray permutation distribution shows GO metric values for 50 METANets with randomly permuted edge scores. METANets significantly outperform both Marbach and PANDA networks and exceed the permutation-based random expectation. (c) GO-directness. For each TF, the fraction of targets annotated with the TF’s most significant GO term that are also supported by ChIP-seq binding evidence, penalizing networks that achieve high GO scores through indirect targets. The gray permutation distribution and black dashed random expectation are as defined in (a) and (b) respectively. METANets significantly outperform both Marbach and PANDA networks. (d) PPI metric. Fraction of the top-ranked (TF, TF) pairs, ranked by Jaccard similarity of their predicted target gene sets, supported by high-confidence PPI in the STRING database (confidence score ≥0.7). The random expectation is the probability that a randomly selected (TF, TF) pair will be supported by PPI data. METANets significantly outperform the Marbach networks at both thresholds. METANets perform comparably to PANDA at the top 50 (TF, TF) pairs and are outperformed by PANDA at the top 100 (TF, TF) pairs, consistent with PANDA’s use of PPI data as a direct input feature.

**Figure 3:**
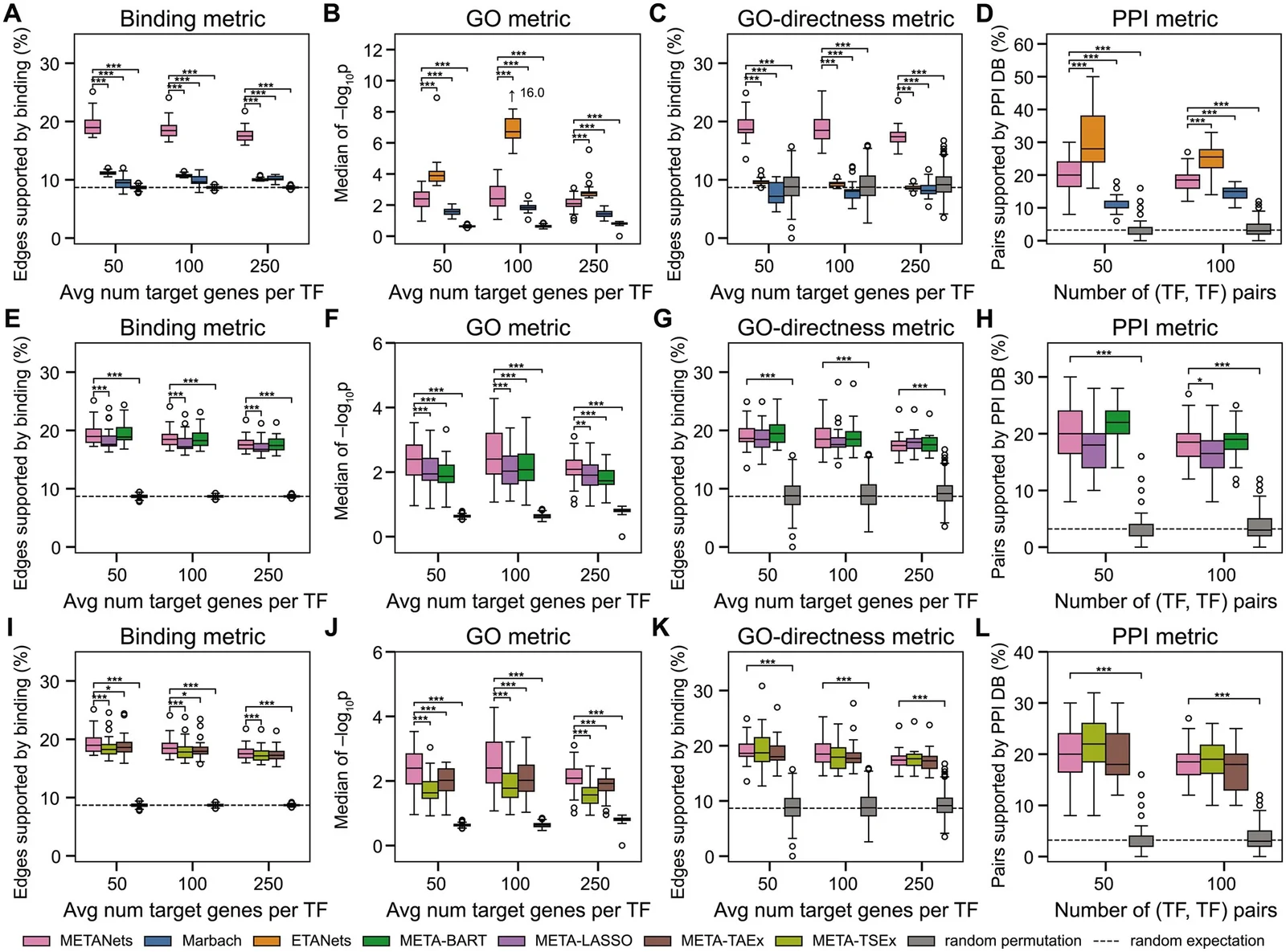
Network quality evaluations against ablated networks. Performance of METANets and ablated network variants evaluated using the four network quality metrics (Section 2.6). Each boxplot shows the distribution of metric values across tissues. Significance bars are drawn only for significant paired *t*-tests with METANets. *P ≤ .05, **P ≤ .01, ***P ≤ .001. Significance levels reflect Benjamini–Hochberg-adjusted *P*-values (see Section 2.6.5). (a–d) Performance of METANets, Marbach networks (motif-only), and ETANets (expression-only) on 36 tissues. (a) Binding metric. METANets significantly outperformed both ETANets and Marbach networks. (b) GO metric. ETANets outperformed METANets and Marbach networks. (c) GO-directness metric. METANets outperformed ETANets, predicting more accurate TF network maps of direct and biologically coherent TF–TG interactions. (d) PPI metric. ETANets outperformed METANets and Marbach networks. (e–h) Performance of METANets and ablated TF network maps META-LASSO and META-BART. (e) Binding metric. METANets and META-BART significantly outperformed META-LASSO, while METANets and META-BART performed comparably. (f) GO metric. METANets outperformed both ablated TF network maps. (g) GO-directness metric. All three TF network maps significantly outperformed the random expectation based on 50 permutations of METANets. METANets are comparable with both ablated network maps. (h) PPI metric. METANets outperformed META-LASSO in the top 100 (TF, TF) pairs only. (i–l) Performance of METANets and ablated TF network maps META-TSEx and META-TAEx. (i) Binding metric. METANets significantly outperformed both ablated TF network maps, except in the top 250 average target genes per-TF threshold, where it was comparable to META-TAEx. (j) GO metric. METANets outperformed both ablated TF network maps. (k) GO-directness metric. All three TF network maps significantly outperformed the random permutation and METANets were comparable with both ablated network maps. (l) PPI metric. All three network maps performed similarly with and outperformed the random permutation.

#### 3.2.2 GO enrichment

To compute the GO metric, we carried out over-representation analysis of GO biological process annotations among the targets of each TF. Each TF’s score was the maximum –log *P*-value over all GO terms. If a TF’s target set showed no significant enrichment for any GO biological process, we assigned the TF a *P*-value of 1 (equivalent to –log *P* = 0). The final network-level score was taken as the median of these scores across all TFs. METANets outperformed Marbach and PANDA (both *P* < .001; Fig. 2b) and surpassed the random permutation (*P* < .001; see Section 2), showing greater biological coherence among the predicted targets of each TF.

#### 3.2.3 GO-directness

The GO-directness metric is the fraction of targets annotated with each TF’s most significant GO term that are also supported by ChIP-seq binding data. This metric penalizes networks that achieve high GO scores by including many, potentially functionally related, but indirect, targets. METANets retained significantly more binding support than both Marbach and PANDA (*P* < .001; Fig. 2c) and the random permutations (*P* < .001).

#### 3.2.4 Protein–protein interaction

The PPI metric is the fraction of top-ranked (TF, TF) pairs, based on target set similarity, that are supported by high-confidence physical PPIs in the STRING database (confidence score ≥ 0.7) (Szklarczyk *et al.* 2023). METANets significantly outperformed Marbach at both thresholds (*P* < .001). Compared to PANDA, METANets performed comparably at the top 50 (TF, TF) pairs (*P* > .05) and were outperformed by PANDA at the top 100 (TF, TF) pairs (*P* < .001) (Fig. 2d). PANDA’s strong performance on this metric is expected, as PANDA incorporates PPI data directly as an input feature in its message-passing framework, providing an inherent advantage when predictions are evaluated against PPI-based evidence. Despite not using PPI data as an input, METANets achieve comparable PPI enrichment at the most stringent threshold and outperform the motif-only Marbach networks, suggesting that the integration of motif and expression features captures complementary information about TF co-regulatory relationships.

Together, these results show that METANets are enriched for direct and likely functional TF–TG interactions relative to other networks. We use the term ‘likely functional’ because METANet incorporates gene expression-derived features as predictors: TF–gene pairs for which a TF’s expression is predictive of a gene’s expression are more likely to represent functionally active regulatory relationships, as gene expression reflects the downstream transcriptional output of regulatory activity. The combination of binding-derived labels and expression-derived input features therefore provides principled, if indirect, evidence that the predicted edges represent not only direct binding events but interactions with functional regulatory consequences.

### 3.3 Combining motif and expression features improves network mapping

We next evaluated the contribution of TF motif- and gene-expression-derived features by comparing METANets to the Marbach networks, which are built using only motifs, and to ETANets, which we built in the same way as METANets but not including the edge score from Marbach networks, using only expression data across all 36 tissues. METANets significantly outperformed both Marbach and ETANet in the Binding metric (*P* < .001), indicating the value of integrating both information sources (Fig. 3a). Interestingly, ETANets performed best in the GO metric, suggesting that gene expression alone captures broader biological coherence (Fig. 3b). However, METANets significantly outperformed ETANets in the GO-directness metric (*P* < .001), indicating that many of ETANets’ additional biologically coherent targets are likely indirect (Fig. 3c). Thus, METANets provide a more accurate map of direct, biologically coherent TF–TG interactions than both the Marbach networks and the PANDA networks.

### 3.4 Linear and nonlinear expression features are synergistic

To assess whether combining linear (LASSO) and nonlinear (BART) expression-derived features improved network quality, we compared METANets against ablated versions using only one feature type: META-LASSO and META-BART. In the Binding metric, METANets and META-BART outperformed META-LASSO. Compared to each other, METANets and META-BART performed comparably (Fig. 3e). In the GO metric, METANets significantly outperformed both ablated network maps (Fig. 3f), suggesting that leveraging both LASSO and BART features better captures biologically coherent relationships. GO-directness was similar across models (Fig. 3g), consistent with gains in biologically coherent targets without loss of direct targets.

### 3.5 Combining tissue-specific and tissue-aggregate expression features is helpful

To assess the contribution of expression-derived feature specificity, we compared METANets to two ablated variants. META-TSEx retains the motif-based Marbach feature and tissue-specific expression features and excludes tissue-aggregate expression features (LASSO and BART trained on all samples pooled across tissues). Conversely, META-TAEx retains the Marbach feature and tissue-aggregate expression features, but not the tissue-specific expression features. In the Binding and GO metrics, METANets outperformed both ablated models (Fig. 3i and j). In the GO-directness metric, METANets significantly outperformed META-TAEx at one threshold (100 targets per TF) (Fig. 3k). Together, these results show that combining both tissue-specific and tissue-aggregate expression features enables METANets to identify substantially more biologically coherent targets of TFs without sacrificing direct binding support.

### 3.6 METANets are tissue specific and comparable to existing networks

We evaluated tissue specificity using tissue-specific expression quantitative trait loci (eQTLs) from GTEx (The GTEx Consortium 2020). For each edge in each tissue METANet, we queried whether at least one tissue-specific eQTL overlapped the TF’s ChIP-seq peaks in the promoter or annotated enhancers of the target gene. We defined two complementary metrics:

1. eQTL support: fraction of edges supported by at least one tissue-specific eQTL.
2. eQTL count: sum, across all edges, of the number of tissue-specific eQTLs supporting each edge.

To assess a network’s tissue specificity, we performed a permutation test. First, we calculated an observed statistic $S_{\mathrm{obs}}$ by ranking all 36 networks within each tissue and summing the ranks of the ‘matching’ pairs (e.g. the rank of the liver network in the liver tissue). A smaller sum indicates better overall specificity. Next, to generate a null distribution, we performed 10 000 permutations. In each permutation, we randomly shuffled the network labels before summing the ranks of the newly assigned matching pairs to obtain the permutation statistic $S_{\mathrm{perm}}$. This process simulates the null hypothesis that there is no true correspondence between a network and its native tissue. The empirical *P*-value was then calculated as the proportion of permutations with $S_{\mathrm{perm}}\leq S_{\mathrm{obs}}.$

### 3.7 METANets are as tissue specific as the Marbach networks

We compared the tissue specificity of top-scoring edges in METANets and the Marbach networks. METANets showed significant tissue specificity compared to the null in both eQTL support and eQTL count (*P* < .05) at higher average targets per-TF thresholds (≥100 targets per TF) (Table 1). The Marbach networks showed similar patterns, and the Wilcoxon signed-rank test revealed no significant difference in the distributions of matching tissue ranks between METANets and Marbach networks in tissue specificity (Table 2). Thus, METANets achieve comparable tissue specificity while outperforming the Marbach networks in all network quality metrics.

**Table 1:** Tissue-specificity evaluation of METANets and Marbach against the null across 26 tissues.

eQTL support	Average number of targets per TF		
	50	100	250
METANets	0.498 (666)	**0.027 (597)**	**0.022 (613)**
Marbach	**0.003 (567)**	**2 × 10^−4^(554)**	**6 × 10^−4^ (537)**

**eQTL counts**	**Average number of targets per TF**		

	**50**	**100**	**250**

METANets	0.106 (604)	**0.003 (535)**	**0.007 (563)**
Marbach	0.119 (611)	0.173 (623)	**3.4 × 10^−3^ (537)**

Permutation test P-values (and the sum of observed matching ranks) against the distribution of 10 000 permutation sums. Bold face indicates *P* < 0.05. See also Fig. S9, available as supplementary data at *Bioinformatics* online.

**Table 2:** Tissue-specificity evaluation against Marbach.

Average numberof targets per TF	50	100	250
eQTL support	0.95 (294.0)	0.30 (266.5)	0.88 (289.0)
eQTL counts	0.24 (243.0)	0.84 (269.5)	0.43 (282.0)

Wilcoxon signed-rank test P-values (and W statistics) between matching tissue ranks of METANets and Marbach networks for 36 tissues.

### 3.8 METANets are comparable to PANDA in tissue specificity but outperform in network quality

Against the PANDA networks (26 tissues), neither PANDA nor METANets showed significant tissue specificity in eQTL support (Table 3). METANets were significantly better than chance in eQTL count at one threshold (average 100 targets per TF), but PANDA was not. The Wilcoxon signed-rank test found no significant difference between METANets and PANDA in either metric, indicating comparable tissue specificity (Table 4).

**Table 3:** Tissue specificity evaluation of METANets and PANDA against the null across 26 tissues.

eQTL support	Average number of targets per TF		
	50	100	250
METANets	0.241 (333)	0.096 (320)	0.143 (324)
PANDA	0.091 (319)	0.176 (330)	0.424 (346)

**eQTL counts**	**Average number of targets per TF**		

	**50**	**100**	**250**

METANets	0.297 (335)	**0.007 (284)**	0.129 (324)
PANDA	0.202 (329)	0.245 (331)	0.183 (328)

Permutation test P-values (and the sum of observed matching ranks) against the distribution of 10 000 permutation sums. Bold face indicates *P* < 0.05. See also Fig. S9, available as supplementary data at *Bioinformatics* online.

**Table 4:** Tissue specificity evaluation against PANDA.

Average number of targets per TF	50	100	250
eQTL support	0.57 (152.0)	0.78 (152.0)	0.87 (156.5)
eQTL counts	0.90 (145.5)	0.41 (132.0)	0.88 (144.5)

Wilcoxon signed-rank test P-values (and W statistics) between matching tissue ranks of METANets and PANDA networks for 26 tissues.

It is important to note that PANDA was originally developed as a general framework for inferring regulatory networks by integrating motif, expression, and PPI data (Glass *et al.* 2013). In its original form, PANDA produces a fully connected, weighted TF–gene network, but does not explicitly model tissue specificity. The concept of *tissue-specific* PANDA networks was introduced by Sonawane *et al*. (2017b), who applied PANDA to 38 GTEx tissues and then post-processed the resulting networks with a tissue-specificity filtering step. In this approach, each edge’s weight in a given tissue was compared to its distribution across all tissues, and a normalized IQR score was used to identify edges unusually strong in one tissue. Edges with high IQR scores were retained as ‘tissue specific’.

To enable direct comparison with the tissue-specific filtering strategy of Sonawane *et al.* we applied the same IQR-based approach to the predicted METANets, producing tissue-specific METANets. These filtered networks were evaluated without thresholding for top-scoring edges, as the number of TFs, genes, and edges varied drastically across tissues. Tissue-specific METANets showed significant eQTL support but not eQTL counts, while tissue-specific PANDA networks showed no significant signal in either metric (Table 5). Again, no significant difference was found between tissue-specific METANets and tissue-specific PANDA networks (Wilcoxon signed-rank *P* > .05) (Table 6).

**Table 5:** Empirical test of tissue-specific METANets and tissue-specific PANDA against the null across 26 tissues.

Empirical test for tissue specificity for 26 tissues		
Network	eQTL support	eQTL count
Tissue-specific METANets	**0.034 (307)**	0.250 (338)
Tissue-specific PANDA	0.233 (332)	0.199 (341)

Wilcoxon signed-rank test P-values (and W statistics) between matching tissue ranks of METANets and PANDA networks for 26 tissues. Bold face indicates *P* < 0.05.

**Table 6:** Tissue specificity evaluation against tissue-specific PANDA.

Wilcoxon signed-rank test versus tissue-specific PANDA	
eQTL support	0.485 (125.0)
eQTL counts	0.881 (168.5)

Wilcoxon signed-rank test P-values (and W statistics) between matching tissue ranks of tissue-specific METANets and tissue-specific PANDA networks for 26 tissues. Full tissue-specific networks were evaluated, without thresholding for top-scoring edges.

Despite similar performance in tissue-specificity, tissue-specific METANets outperformed tissue-specific PANDA in the Binding and GO-directness metrics (*P* < .05 and *P* < .001, respectively), while performing similarly in the GO metric (Fig. 4a–c). Tissue-specific METANets also performed better in the PPI metric overall (Fig. 4d).

**Figure 4:**
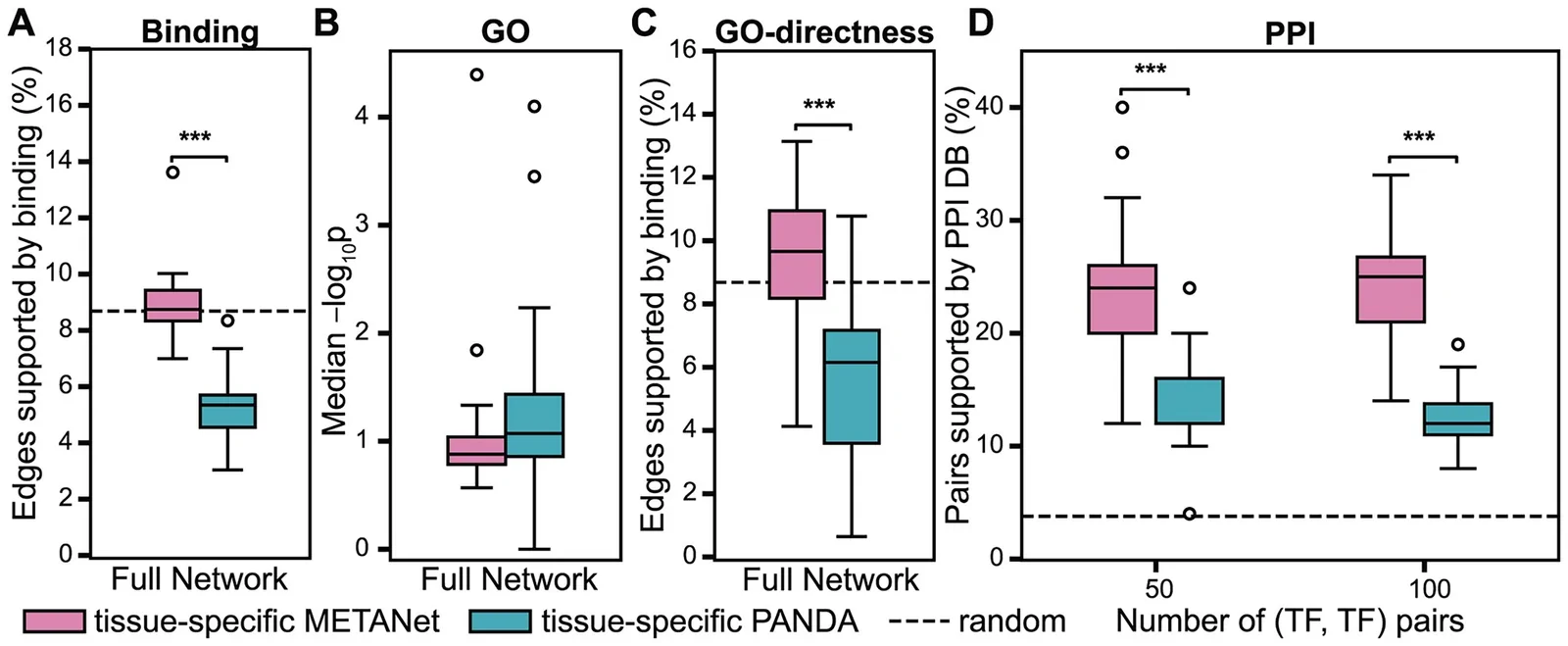
(a–d) Network quality evaluation of tissue-specific METANets and tissue-specific PANDA networks. (a) Binding metric. Tissue-specific METANets significantly outperformed tissue-specific PANDA networks (paired *t*-test, *P* < .05). (b) GO metric. No significant difference between tissue-specific METANets and tissue-specific PANDA networks. (c) GO-directness metric. Tissue-specific METANets outperformed tissue-specific PANDA networks (*P* < .001). (d) Tissue-specific METANets outperformed tissue-specific PANDA networks at thresholds of 50 and 100 (TF, TF) pairs. *P ≤ .05, **P ≤ .01, ***P ≤ .001.

### 3.9 Tissue aggregation of binding labels does not distort network evaluation

To determine the effects of tissue-aggregated binding labels, we evaluated METANets and PANDA networks for whole blood, liver, and breast mammary tissue against tissue-specific binding labels derived from three cell lines (K-562, Hep-G2, and MCF-7) as well as against the aggregate labels (Fig. 5a). METANets (AUROC ∼0.58–0.60) outperformed PANDA networks (AUROC ∼0.52–0.55) against every label set in all three tissues (paired Wilcoxon signed-rank test on shared TFs, all *q* < 0.015), confirming that the advantage of the METANet framework does not depend on the use of aggregated labels. Moreover, performance against the aggregate labels closely tracked performance against the tissue-matched cell line labels, indicating that aggregation does not distort the benchmark.

**Figure 5:**
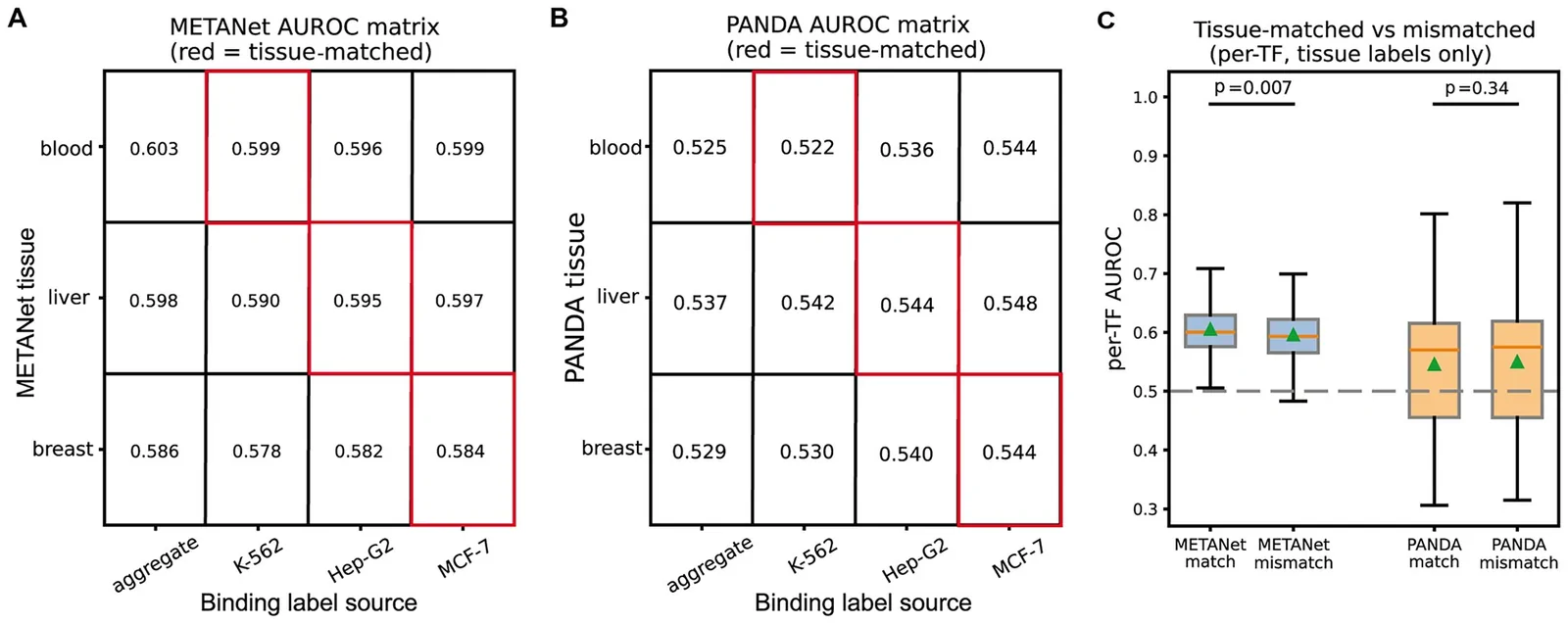
Evaluation against cell-line-specific binding labels. METANet and PANDA networks for whole blood, liver, and breast mammary tissue were evaluated against binding labels from three matched cell lines (K-562, Hep-G2, MCF-7) and against the tissue-aggregated labels (Section 2.7.3). (a) Per-TF AUROC of METANets across networks (rows) and binding label sets (columns); In the boxed diagonal, the network tissue and the label tissue match. (b) Same as (a) for PANDA networks. (c) Distribution of per-TF AUROC for METANets on tissue-matched versus tissue-mismatched cell line labels, across tissues; the matched distribution is significantly higher for METANet (one-sided Mann–Whitney *U* test, *P* = .007), but not for PANDA. Green triangles indicate means and the central line indicates the median.

We next tested whether each tissue network performed best on its tissue-matched cell line. Across the three tissues, METANets achieved higher AUROC on tissue-matched than on tissue-mismatched cell line labels (median 0.600 versus 0.593; one-sided Mann–Whitney *U* test, *P* = .007; Fig. 5c), whereas PANDA networks showed no such preference (*P* = .34). This indicates that the METANets encode a modest, but statistically detectable tissue-specific binding signal that PANDA does not. At the level of individual tissues, the difference between matched and mismatched tissues was only nominally significant for whole blood and liver (paired Wilcoxon test, BH-adjusted  q= .034 and q = .046, respectively). We therefore interpret the tissue-matching signal as real in aggregate, but too small to attribute to any single tissue pair. Refer to Fig. S10, available as supplementary data at *Bioinformatics* online, for additional information.

Together with the eQTL-based analysis (Sections 3.6–3.8), these results indicate that METANets carry tissue-specific regulatory information, while confirming that the tissue-aggregated benchmark provides a fair basis for the network comparisons reported above.

### 3.10 Tissue-specific METANets are useful for genetic analyses

To evaluate the biological relevance of tissue-specific METANets, we applied FISHNET, a method that integrates gene-level *P*-values with network modules and functional annotations to identify genes with suggestive association signals that are more likely to replicate across independent datasets (Acharya *et al.* 2025). Using the whole blood tissue-specific METANet as the reference network, we provided gene-level *P*-values derived from measured TWAS of 11 traits associated with cardiovascular risk in the LLFS cohort (Acharya *et al.* 2024) and performed replication analysis in the FHS cohort (Kannel *et al.* 1979, Splansky *et al.* 2007). Across the 11 traits, FISHNET identified 114 FISHNET genes, of which 24 were replicated. Using the whole blood tissue-specific PANDA network as input, we identified 0 FISHNET genes (see Tables S6 and S7, available as supplementary data at *Bioinformatics* online).

A central replicated finding was *SREBF2*, negatively associated with high-density lipoprotein (HDL). In the whole blood tissue-specific METANet, *SREBF2* co-localized in a module with *S100A9, S100A12, TLR8*, and *ITGB2—*genes converging on inflammatory and oxidative stress pathways that link cholesterol metabolism to chronic low-grade inflammation. This regulatory architecture suggests a mechanistic connection between reduced HDL, *SREBF2* activation, and sustained inflammatory signaling. Full biological interpretation of this finding is provided in the Supplementary Methods, available as supplementary data at *Bioinformatics* online.

## 4 Discussion

In this work, we developed the METANet framework, a supervised ensemble approach that combines TF motifs, CRE activity, and expression-derived features to construct tissue-specific TF network maps across 36 human tissues. Our modeling approach relied on three fundamental ideas: (1) using gene expression data (functional) to predict TF binding events (direct) prioritizes direct, likely functional targets of TFs, (2) incorporating both tissue-aggregate and tissue-specific expression features captures both the shared and tissue-specific regulatory edges, and (3) using tree-based regression models to incorporate both linear and nonlinear expression patterns improves model robustness. Our evaluations across multiple orthogonal metrics show that our approach significantly outperforms motif-only, expression-only, and unsupervised integrative methods in capturing direct and biologically coherent TF–TG relationships.

Gene-expression-based models are adept at identifying likely functionally coherent gene sets, but the expression of a TF and a potential target gene could be correlated for reasons other than the TF regulating the gene directly: the TF might regulate indirectly (mediated by other proteins), or the TF and the gene might simply be co-regulated by a third factor. Conversely, motif-based approaches anchor predictions in physical binding potential but cannot confirm functional relevance, as potential binding sites are frequently unoccupied (Wang *et al.* 2012, Dror *et al.* 2015) and even occupied sites often do not affect transcription (White *et al.* 2013, Cusanovich *et al.* 2014, Slattery *et al.* 2014, Kang *et al.* 2020, Mahendrawada *et al.* 2025). By training our models to explicitly predict TF ChIP-seq binding events, the METANet framework leverages the strengths of both data types without a noticeable trade-off. Our results demonstrate that the resulting predicted METANet maps (METANets) consistently outperform the motif-only Marbach networks and our expression-only ETANets in the GO-directness metric that jointly assesses directness and biological coherence.

The METANet framework also illustrates the power of incorporating diverse feature types to model the complexity of gene regulation. We found that regulatory relationships are not exclusively linear or nonlinear; by combining features from both LASSO (linear) and BART (nonlinear) regression models, METANets captured a more direct and biologically coherent set of TF targets. Similarly, we found that neither tissue-specific nor tissue-aggregate features clearly outperformed the other in isolation, but together they predicted more robust sets of TF targets. The METANet approach performed best in the individual Binding and GO metrics without sacrificing GO-directness as a trade-off. The practical utility of this approach was highlighted in our downstream genetic analysis, where the whole blood METANet enabled FISHNET to identify replicated gene–trait associations, proposing a module linking *SREBF2* to HDL and chronic inflammation.

Our work presents a subtle methodological shift for the field of regulatory network inference. For years, the community has largely relied on two distinct categories of methods: those based on physical binding evidence like TF motifs, which often lack functional validation, and those based on gene expression, which are susceptible to confounding by indirect relationships. While unsupervised integration methods have attempted to bridge this gap, the METANet approach presents a new alternative by successfully implementing a supervised learning framework. The core innovation is reframing the problem: instead of merely correlating different data types, we use functional data (gene expression) to predict direct physical interactions (TF binding).

This approach provides a methodological foundation for using the ever-expanding space of omics data integration. The principle of using one data modality as a ground-truth label to train predictive models on other, more widely available data types can be generalized beyond the specific application in this work. It makes TF network mapping more broadly applicable, as ChIP-seq data for every TF is not available, while RNA-seq data is abundant. Our approach can make use of ChIP-seq in predicting targets of TFs that have ChIP-seq data, but it can also extract general patterns in gene expression data that are predictive of direct binding and use those patterns to infer targets for TFs that do not have ChIP-seq data. We also presented a new evaluation of networks based on utility for applications rather than network characteristics.

Several limitations suggest avenues for further development. One key limitation is the lack of tissue-specific TF binding data in our framework. While tissue-specific expression data are readily available at scale, obtaining comparable TF binding data across tissues remains challenging. As a result, the METANet framework relied on tissue-aggregated TF binding data. Consequently, METANets are best interpreted as context-informed predictions, with tissue specificity conferred by tissue-specific expression and CRE activity input features, rather than as directly supervised inference of tissue-specific TF binding. A direct evaluation against cell line-specific labels (Fig. 5) indicated that aggregation does not grossly distort the benchmark. Emerging paired-seq technologies that jointly measure TF binding and gene expression in the same cell could make predictions of TF binding events more robust, and the single-cell measurements can facilitate the direct mapping of granular cell-type-specific networks. Development of scalable methods to address the inherent sparsity of single-cell data will be crucial for realizing this goal.

Another opportunity lies in extending the METANet framework to additional omics data types. Chromatin accessibility (ATAC-seq) and chromatin conformation (Hi-C, HiChIP) represent rich sources of contextual information that could further refine predictions of TF–TG relationships. Beyond tissues, the same framework could be adapted to disease states, environmental exposures, or dynamic perturbations, producing condition-specific network maps that illuminate regulatory mechanisms underlying phenotypic variation.

In summary, the METANet framework advances the state of regulatory network mapping by demonstrating that supervised integrative approaches can accurately prioritize direct, likely functional TF–TG interactions. Beyond their immediate utility, this approach highlights the potential of combining heterogeneous data sources to produce context-dependent regulatory maps, setting the stage for increasingly precise models of human gene regulation.

## Supplementary Material

btag738_Supplementary_Data

## Data Availability

All data and code are available at https://doi.org/10.5281/zenodo.20614479.
