# Supplementary material for: METANet: a supervised ensemble learning framework for reconstructing direct and functional tissue-specific transcription factor networks
Source: Bioinformatics. 2026 Oct 7;42(10):btag738. doi: 10.1093/bioinformatics/btag738 (PMC13693595; doi:10.1093/bioinformatics/btag738)
Supplement: Supplementary file 1 [file btag738_supplementary_data.zip › Supplementary Material final.pdf]

Supplementary Material for "METANet: A supervised ensemble learning framework for reconstructing direct and functional tissue-specific transcription factor networks"

Wooseok J. Jung[1,2], Sandeep Acharya[2,3], Daniel P. Ruskin[4], Shu Liao[1,2], Vaha Akbary Moghaddam[5], Zolboo Erdenebaatar[1,2], Michael R. Brent[1,2,6,*]

[1]Department of Computer Science and Engineering, Washington University, St Louis, MO,

[2]Center for Genome Sciences and Systems Biology, Washington University School of Medicine, St. Louis, MO,

[3]Division of Computational and Data Sciences, Washington University, St Louis, MO,

[4]College of Information, University of Maryland, College Park, MD,

[5]Division of Statistical Genomics, Washington University School of Medicine, St Louis, MO,

[6]Department of Genetics, Washington University School of Medicine, St. Louis, MO

*To whom correspondence should be addressed

Michael R. Brent

brent@wustl.edu

(314) 362-7238

Fax: (314) 362-2156

Campus Box 8510

Washington University

Saint Louis, MO 63130

Table of Contents

Supplementary Figure S1 3

Supplementary Figure S2 4

Supplementary Figure S3 4

Supplementary Figure S4 5

Supplementary Figure S5 5

Supplementary Figure S6 6

Supplementary Figure S7 7

Supplementary Figure S8 8

Supplementary Figure S9 9

Supplementary Figure S10 10

Supplementary Methods 11

- **Source and aggregation of ChIP-seq binding data** 11
- **Mapping binding peaks to regulatory regions** 11
- **Construction Of Binding Labels** 11
- **Processing of PANDA Networks** 11
- **Tissue-specific network filtering** 12
- **Validation of Tissue Specificity using eQTL Data** 12
- **Tissue-specific METANets are Useful for Genetic Analyses** 13

Supplementary Figure S1

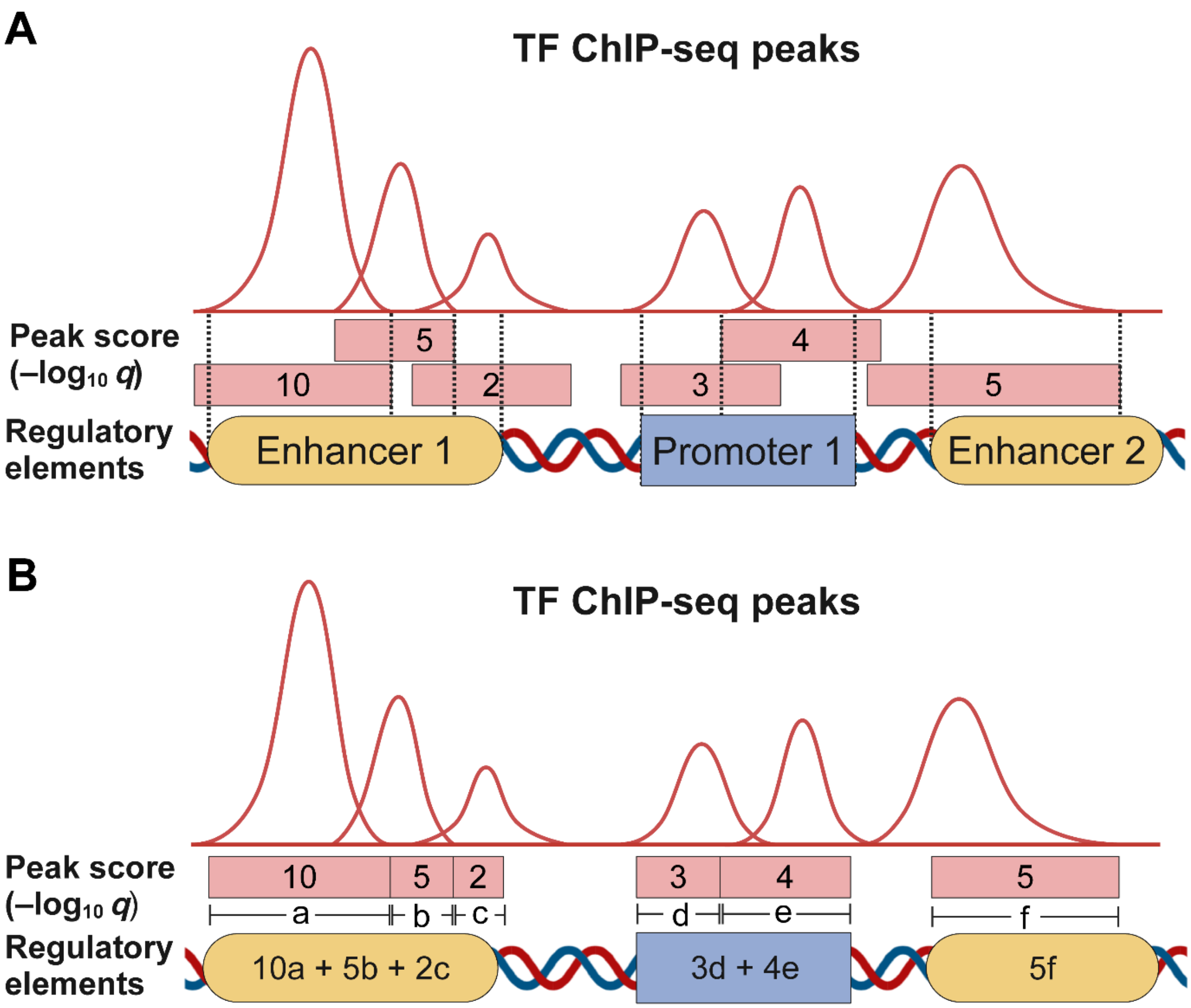

Supplementary Figure S1 Example illustration of assigning TF binding scores to FANTOM5 regulatory elements. (A) Multiple TF ChIP-seq peaks may overlap in a single regulatory element. Dotted vertical lines indicate cutoffs for overlapping peaks, retaining the peak with maximum peak score. (B) Each regulatory element's score is calculated as the sum of retained peak scores weighted by the peak region length.

Supplementary Figure S2

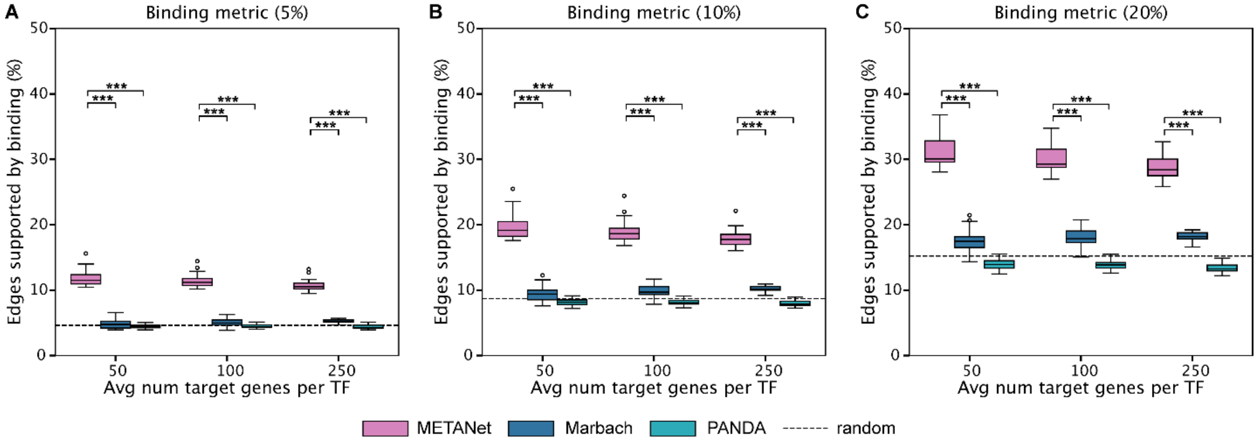

Supplementary Figure S2 Robustness of Binding metric performance across binding label definitions Binding metric evaluated at 5% (A), 10% (B), and 20% (C) binding label thresholds. For each threshold, the fraction of predicted TF-gene edges supported by binding evidence is shown for METANet, Marbach networks, and PANDA networks across 26 GTEx tissues, at network sizes corresponding to 50, 100, and 250 average targets per TF. The binding label threshold defines a per-TF ceiling on the maximum number of positive labels, set at the specified percentage of the gene universe. TFs with fewer binding-supported targets than this ceiling retain all their positive labels without truncation; consequently, the actual positive label rate across all TF-gene pairs is lower than the nominal threshold (~4.6%, ~8.7%, and ~15.2% at the 5%, 10%, and 20% thresholds respectively). The dashed horizontal line indicates the random expectation computed from the actual positive label rate under each respective threshold and thus differs across panels. METANet outperforms both Marbach and PANDA networks at all three threshold definitions, demonstrating that binding prediction performance is robust to the choice of binding label threshold. *P≤0.05, **P≤0.01, ***P≤0.001.

Supplementary Figure S3

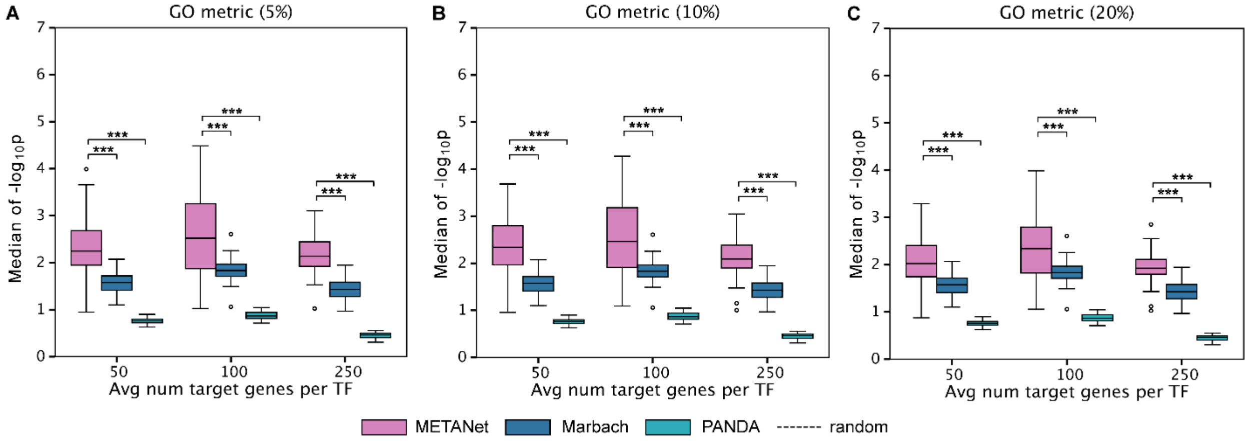

Supplementary Figure S3 Robustness of GO metric performance across binding label thresholds GO metric evaluated at 5% (left), 10% (center), and 20% (right) binding label thresholds for METANet, Marbach networks, and PANDA networks across 26 GTEx tissues, at network sizes corresponding to 50, 100, and 250 average targets per TF. As the GO metric is based on gene ontology annotations independent of binding label definitions, results are highly consistent across thresholds. METANet outperforms both PANDA and Marbach networks at all three threshold definitions. *P≤0.05, **P≤0.01, ***P≤0.001.

Supplementary Figure S4

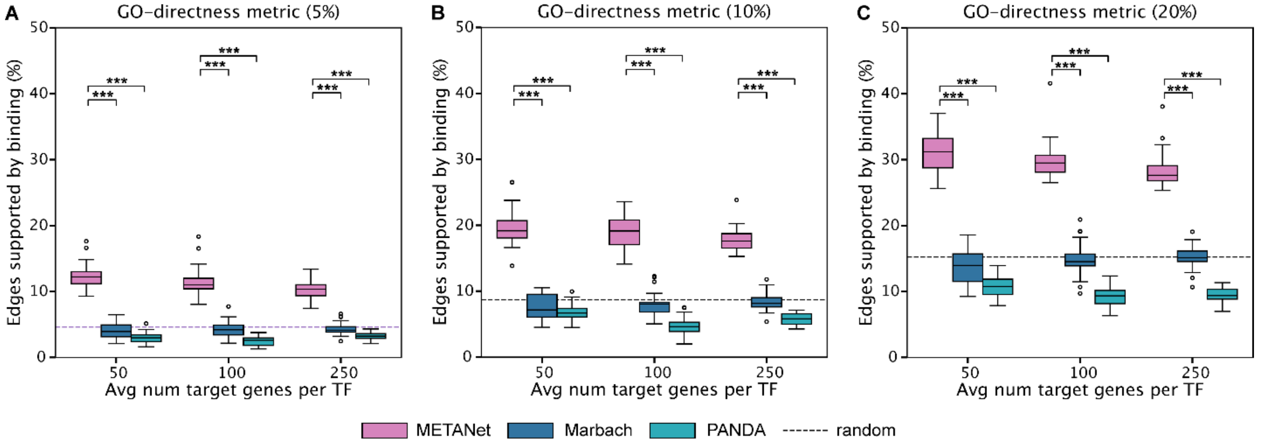

Supplementary Figure S4 Robustness of GO-directness metric performance across binding label thresholds GO-directness metric evaluated at 5% (left), 10% (center), and 20% (right) binding label thresholds for METANet, Marbach networks, and PANDA networks across 26 GTEx tissues, at network sizes corresponding to 50, 100, and 250 average targets per TF. As the GO-directness metric incorporates binding label definitions to assess the specificity of TF-target relationships, absolute performance values differ across thresholds consistent with the differing positive label rates. Nevertheless, METANet outperforms both PANDA and Marbach networks at all three threshold definitions, demonstrating that the relative advantage of METANet is robust to the choice of binding label threshold. *$P \leq 0.05$, **$P \leq 0.01$, ***$P \leq 0.001$.

Supplementary Figure S5

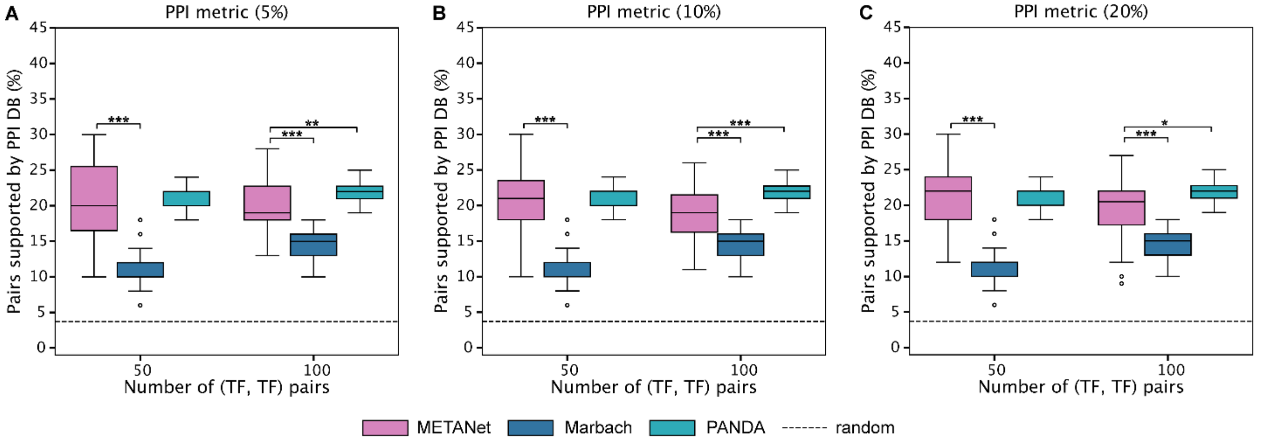

Supplementary Figure S5 Robustness of PPI metric performance across binding label thresholds PPI metric evaluated at 5% (left), 10% (center), and 20% (right) binding label thresholds for METANet, Marbach networks, and PANDA networks across 26 GTEx tissues. As the PPI metric relies on an external protein-protein interaction database independent of binding label definitions, results are highly consistent across thresholds. PANDA consistently outperforms METANet on this metric at the top 100 (TF, TF) pairs by target gene set similarity. PANDA incorporates PPI data directly as an input feature in a message-passing framework to construct its regulatory networks, providing it with an advantage on PPI-based evaluation. METANet, which does not use PPI data as an input, achieves comparable performance at the top 50 (TF, TF) pairs and outperforms the Marbach networks at all binding threshold definitions. *$P \leq 0.05$, **$P \leq 0.01$, ***$P \leq 0.001$.

Supplementary Figure S6

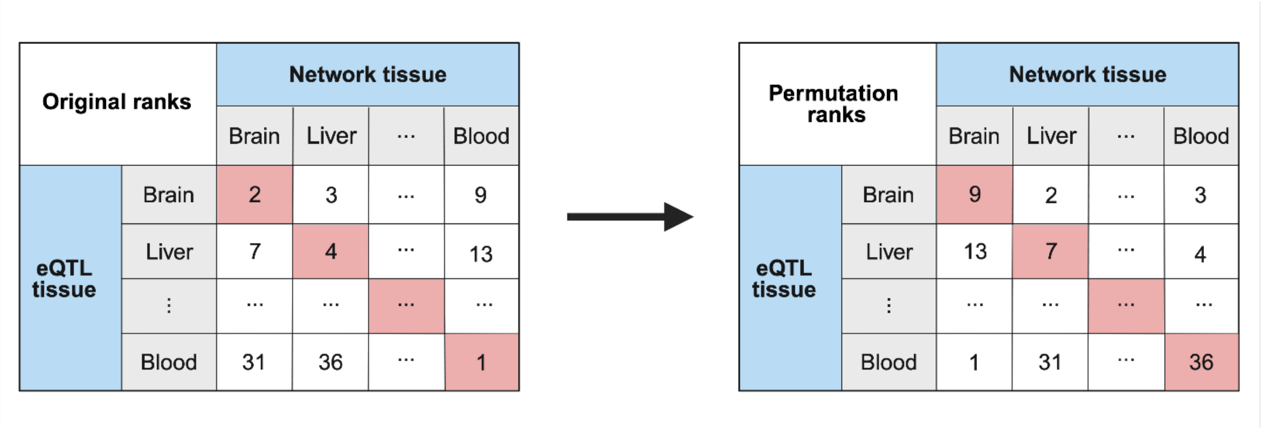

Supplementary Figure S6 Permutation to generate empirical null distribution of ranks For each tissue's eQTL data, all tissue networks are ranked based on either the eQTL support or eQTL counts metric, breaking ties using the other metric. Each diagonal entry in the original ranks table represents the rank of the "matching" network for the given tissue's eQTL. The sum of the diagonal entries in the original ranks table (left) is the observed statistic. For each of 10000 permutations, we shuffle the columns of the table. The sum of the diagonal entries in this shuffled table is the permuted statistic. This process simulates the null hypothesis that there is no true correspondence between a network and the tissue it was constructed for.

Supplementary Figure S7

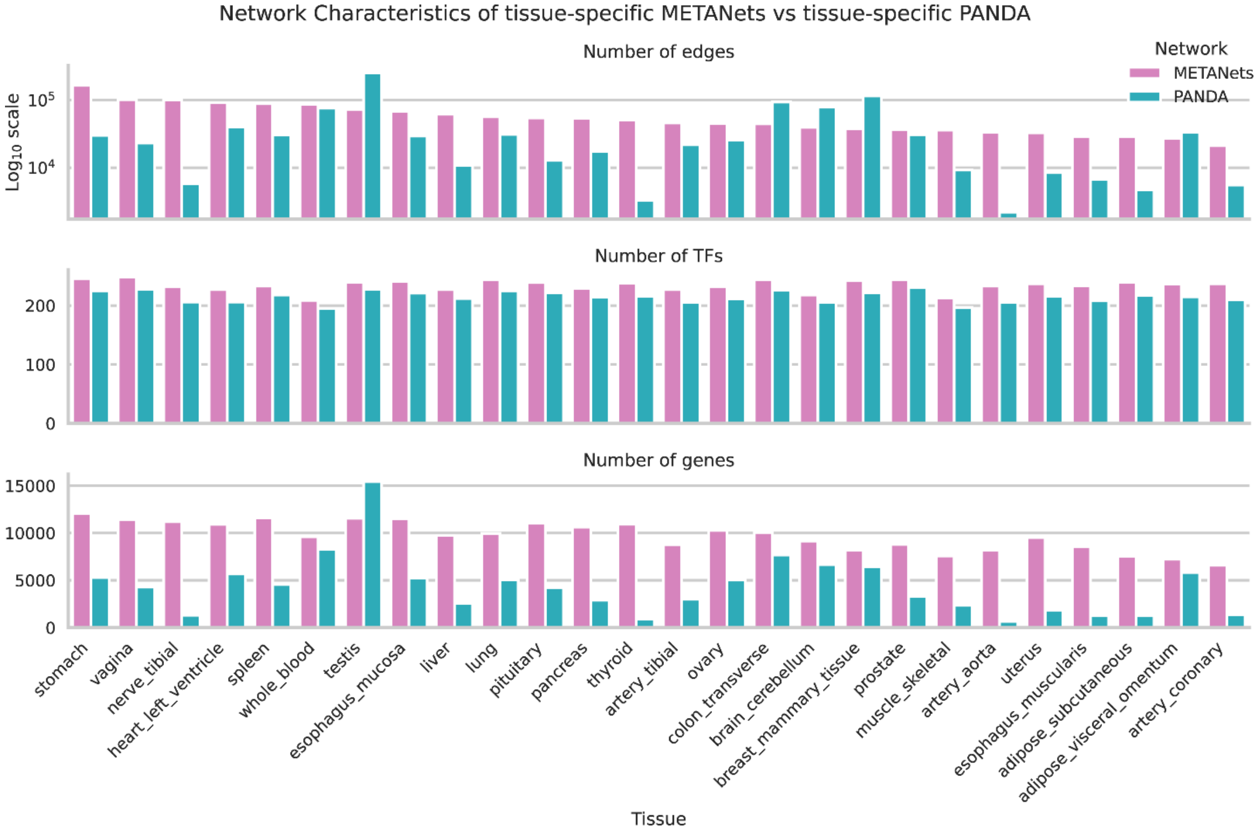

Supplementary Figure S7 Network characteristics of tissue-specific METANets and tissue-specific PANDA networks PANDA networks were first restricted to protein-coding genes, with self-regulatory edges excluded, and subsequently subsetted to TFs present in the corresponding tissue's METANet. See Supplementary Table S2 for tabulated details of this figure.

Supplementary Figure S8

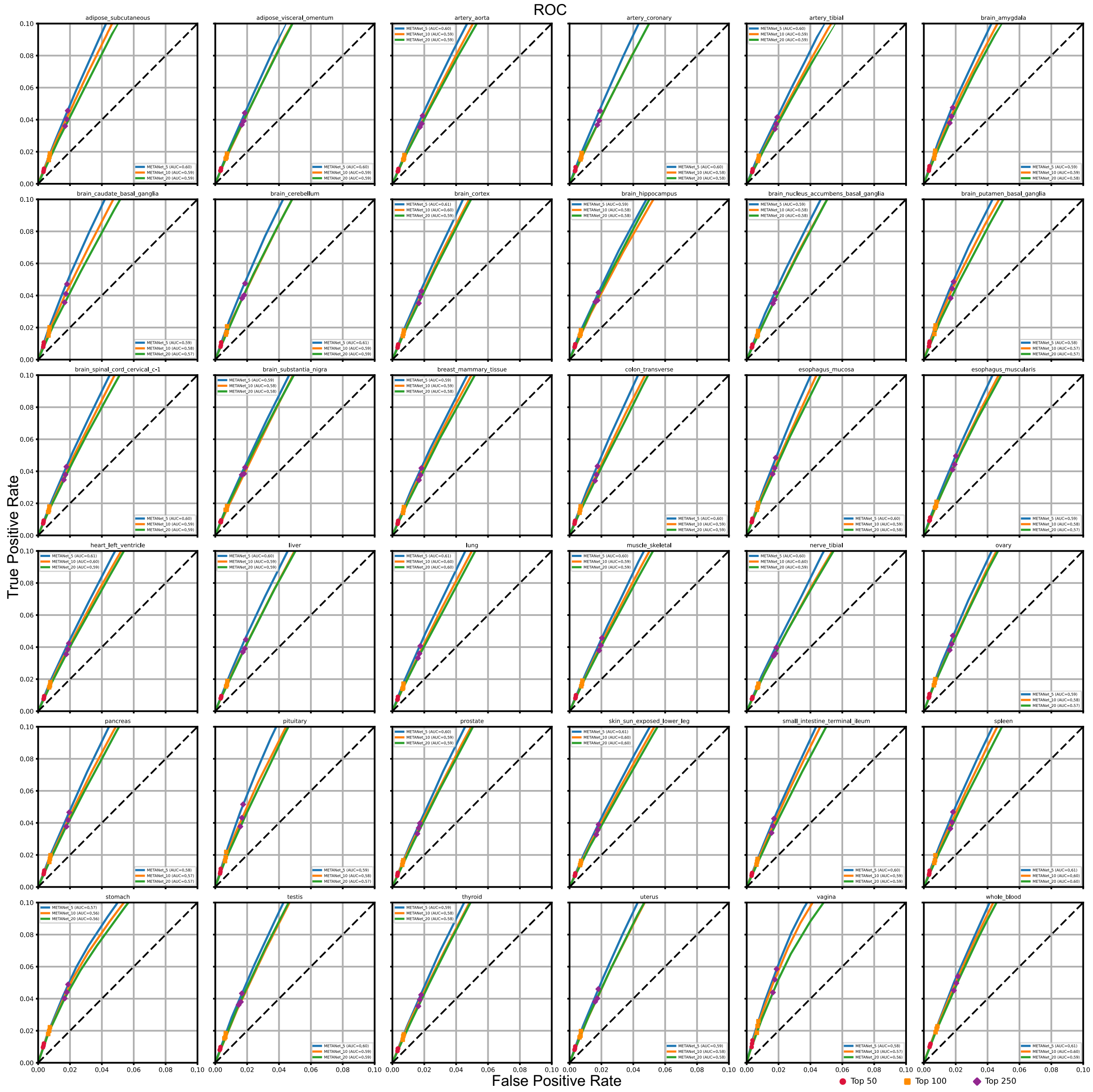

Supplementary Figure S8 ROC curves for METANet TF binding predictions across binding label thresholds and network size thresholds Receiver operator characteristic (ROC) curves for METANets evaluated across 36 GTEx tissues. ROC curves and AUROC values are computed using the full network of predicted TF-gene edges. For each tissue, three ROC curves are shown corresponding to binding labels defined at 5% (blue), 10% (orange), and 20% (green) binding thresholds, with AUROC values annotated in the subplot legend. Three operating points are annotated on each curve corresponding to the network size thresholds used in the main network quality evaluation figures: the top 50, 100, and 250 average targets per TF. To ensure these operating points are clearly visible, subplot axes are restricted to [0, 0.1] on both axes; the full ROC curves extend to [1, 1] but are not shown as the region of interest corresponds to the high-confidence top-scoring edges. The diagonal dashed line indicates random classifier performance within the displayed axis range. ROC curves across the three binding threshold definitions are largely overlapping within each tissue, with minimal differences in AUROC, demonstrating that the discriminative performance of METANet predictions is robust to the choice of binding label threshold.

Supplementary Figure S9

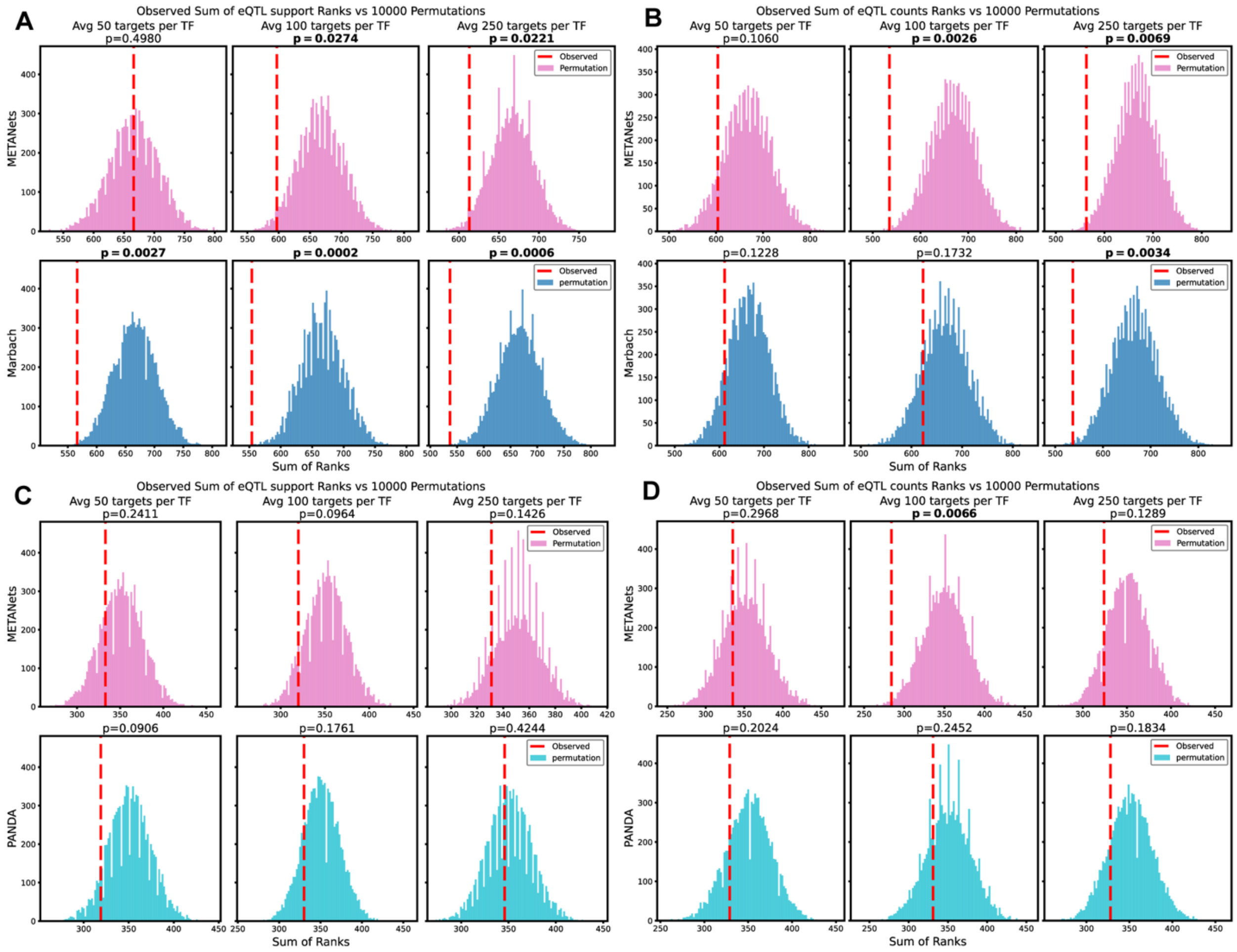

Supplementary Figure S9 Tissue specificity evaluation against the empirical null. The sum of eQTL support or eQTL counts ranks (dashed red line) plotted against a null distribution of 10000 sums of permuted ranks. METANets and Marbach networks were evaluated across 36 tissues for (**A**) eQTL support and (**B**) eQTL counts. METANets and PANDA networks were evaluated across 26 tissues for (**C**) eQTL support and (**D**) eQTL counts. METANets have tissue specificity comparable to other networks, except that Marbach networks have better eQTL support at average 50 targets per TF (panel A).

Supplementary Figure S10

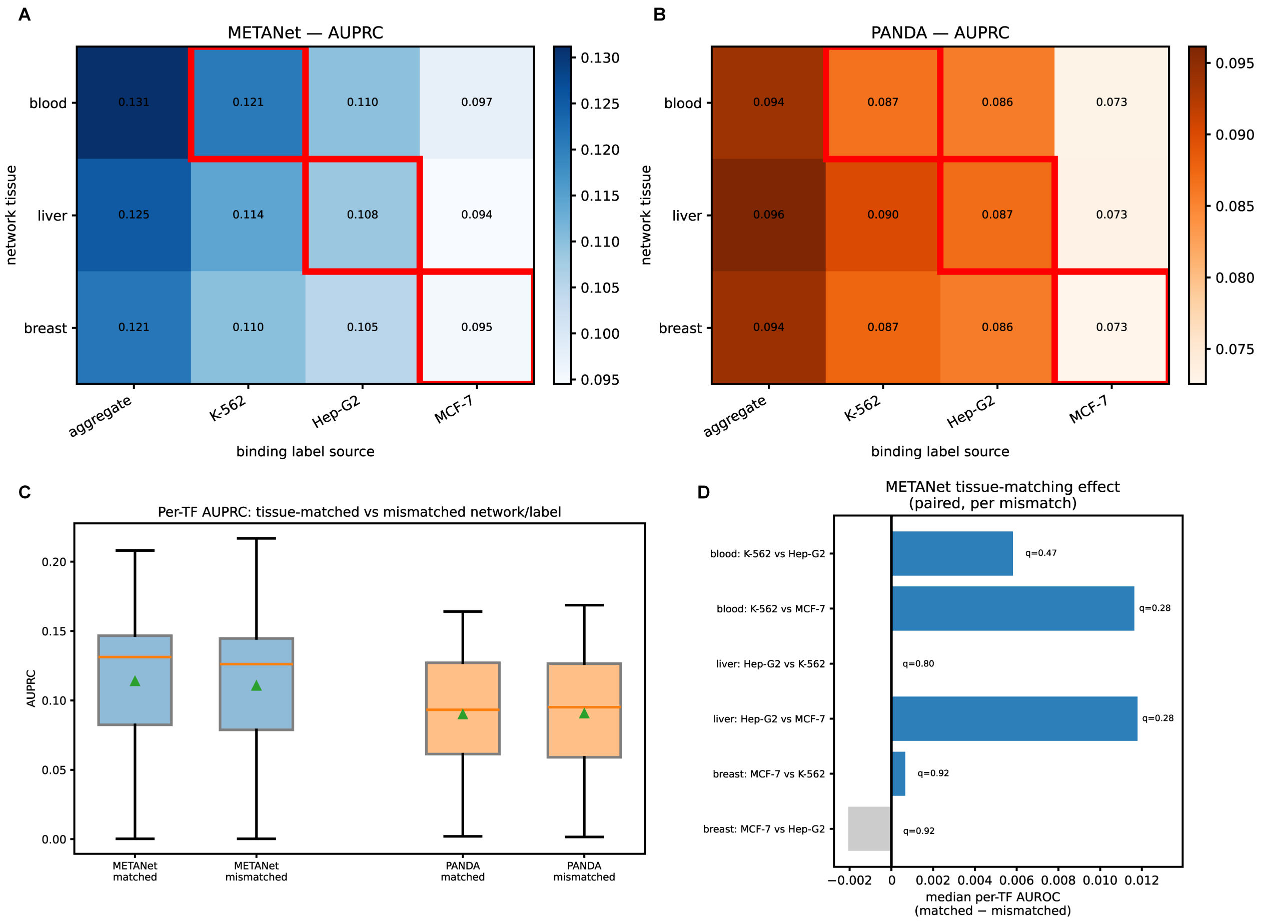

Supplementary Figure S10 Cross-tissue binding evaluation: AUPRC matrices and supporting comparisons. Companion to Fig. 5. METANet and tissue-specific input PANDA predictions for whole blood, liver, and breast mammary tissue were evaluated against the tissue-aggregated binding labels and against cell-line-specific labels from three matched cell lines (K-562, Hep-G2, MCF-7); see Section 2.7.3. (**A,B**) Per-TF AUPRC of (A) METANets and (B) PANDA networks across tissue networks (rows) and binding-label sets (columns); tissue-matched network-cell-line pairs lie on the boxed diagonal. Because AUPRC scales with positive-class rate, which differs across label sets (e.g. ~8.9% for the aggregate labels vs. ~6.6% for MCF-7), AUPRC values are not directly comparable across columns, and AUROC (Fig. 5) is therefore used as the primary cross-tissue metric. (**C**) Distribution of per-TF AUPRC for METANets on tissue-matched vs. tissue-mismatched cell-line labels, across tissues; green triangles indicate means and the central line indicates the median. In contrast to AUROC, the matched and mismatched distributions do not differ significantly (one-sided Mann-Whitney U test, $p \approx 0.15$), consistent with AUPRC reflecting prevalence rather than tissue-matched binding. (**D**) Median per-TF AUROC (x-axis) for METANets on tissue-matched vs. tissue-mismatched cell-line labels, summarizing the small but significant matched > mismatched shift quantified in Fig. 5C (one-sided MWU test, $P=0.007$).

Supplementary Methods

Source and aggregation of ChIP-seq binding data

TF binding data were obtained from the REMAP 2020 database (Cheneby et al., 2020), which provides individual ChIP-seq peak files for each experiment. For each TF in our network, all available ChIP-seq experiment files from REMAP 2020 were downloaded. The peaks across all retained experiments for a given TF were then concatenated into a single set and filtered for high-confidence peaks (FDR q-value ≤ 0.01). This pooled, filtered peak set was then mapped to FANTOM5 regulatory elements as described below. The resulting gene-level binding scores therefore represent binding evidence aggregated across primary tissue ChIP-seq experiments, where aggregation across experiments is performed by taking the union of peaks prior to regulatory element mapping, and overlapping peaks within a single regulatory element are resolved by retaining the highest-confidence peak.

Mapping binding peaks to regulatory regions

For each TF, all high-confidence (FDR q-value ≤ 0.01) binding peaks within FANTOM5 promoter and enhancer regions were examined (Andersson, et al., 2014; The GTEx Consortium, 2020). In cases where multiple overlapping peaks were present within a single regulatory element, the peak with the highest confidence score among the overlapping regions was retained. For each nucleotide within a FANTOM5 promoter or enhancer region, a score was calculated as the maximum -log10(q-value) across all peaks overlapping that nucleotide (see Supplementary Fig. S1). For each gene, a gene score was derived by aggregating the scores of all regulatory elements annotated for the gene. Since TF binding events within each regulatory region represent independent evidence of gene regulation, the aggregated score across regulatory elements reflects the cumulative likelihood of the TF regulating the gene.

Construction Of Binding Labels

The network mapping approach uses binary labels representing binding events for each TF-gene pair. We assigned a positive label to genes with nonzero binding scores for each TF to construct binary labels for TF-gene binding events. If more than 10% of genes had a positive score for a given TF, only the top 10% of highest-scoring genes were retained as positive labels. All remaining gene pairs were labeled as negative.

Processing of PANDA Networks

For benchmarking, we downloaded PANDA networks for 38 human tissues from Zenodo https://doi.org/10.5281/zenodo.838734 (Sonawane, 2017). To enable direct comparison with our METANets, we retained 26 tissues shared with the 36 METANet tissues. For each tissue, we used two

versions of the PANDA networks: (1) the full networks containing weighted edges between all transcription factors (TFs) and genes, and (2) the tissue-specific networks provided in the same dataset, in which tissue-specific edges had already been annotated by the original authors. To maintain consistency, both versions of the PANDA networks were subjected to the same downstream processing steps: restricting to protein-coding gene targets, removing self-regulatory edges, and retaining only the subset of TFs common to both PANDA and METANets.

Tissue-specific network filtering

Tissue-specific regulatory edges were identified using the filtering approach originally introduced by Sonawane et al. (Sonawane, et al., 2017). For each TF-TG pair *(i,j)*, the edge weight in tissue *t* is denoted $w_{ij}^{(t)}$. For each edge, we computed a normalized specificity score by comparing the tissue-specific weight to the distribution of weights across all tissues:

$$s_{ij}^{(t)} = \frac{w_{ij}^{(t)} - \text{median}(w_{ij})}{\text{IQR}(w_{ij})},$$

where $\text{median}(w_{ij})$ and $\text{IQR}(w_{ij})$ are calculated across all tissues for the same edge. Edges with $s_{ij}^{(t)} > 2$ were considered specific to tissue *t*. This filtering approach was applied to METANets to construct tissue-specific versions of our networks, directly comparable to the tissue-specific PANDA networks generated in Sonawane et al.

Each METANet map varies in total edge count due to differences in gene expression coverage; some genes fail to pass tissue-specific expression filters and thus lack corresponding edges in certain tissues. Because the tissue-specificity score $s_{ij}^{(t)}$ depends on computing both the median and the IQR of edge weights w_{ij} across all tissues, edges missing in many tissues results in too few observations to reliably estimate their across-tissue variability. We therefore excluded edges absent in more than 12 of the 36 tissues. This step retained 77.16% of the complete edge set.

Validation of Tissue Specificity using eQTL Data

We downloaded cis-eQTL data from the GTEx consortium V8 release (The GTEx Consortium, 2020). Significant variant-gene associations were obtained for each of the 36 tissues. For each TF-target gene (TF-TG) edge in a network, we determined whether one or more of a tissue's significant variants overlapped the TF's ChIP-seq peaks within the promoter or annotated enhancers of the target gene. We defined two complementary metrics for a given network:

1. eQTL support: the fraction of edges supported by at least one tissue-specific eQTL.

2. eQTL count: the sum, across all edges, of the number of tissue-specific eQTLs supporting each edge.

To assess a network's tissue-specificity, we performed a permutation test. First, we calculated an observed statistic S_{obs} by ranking all 36 networks within each tissue and summing the ranks of the "matching" pairs (e.g., the rank of the liver network in the liver tissue). A smaller sum indicates better overall specificity. Next, to generate a null distribution, we performed 10000 permutations. In each permutation, we randomly shuffled the network labels before summing the ranks of the newly assigned matching pairs to obtain the permutation statistic S_{perm}. This process simulates the null hypothesis that there is no true correspondence between a network and its native tissue. The empirical p-value was then calculated as the proportion of permutations with $S_{perm} \leq S_{obs}$ (see Supplementary Figure S6).

Direct comparisons between different networks were performed using the Wilcoxon signed-rank test on the distributions of matching ranks.

Tissue-specific METANets are Useful for Genetic Analyses

To evaluate the biological relevance of tissue-specific METANets, we applied FISHNET, a method that uses prior biological knowledge to identify genes with suggestive association signals that are more likely to replicate across independent datasets (Acharya, et al., 2025). FISHNET integrates gene-level P-values with networks and functional annotations, based on the principle that spurious signals are randomly scattered, while true signals tend to cluster in functionally coherent subnetworks. Combining network topology, functional enrichment, and permutation-derived thresholds, FISHNET identifies a focused set of exceptional candidates termed FISHNET genes.

The primary input to FISHNET was the whole blood tissue-specific METANet. For comparison, we also applied FISHNET using the whole blood tissue-specific PANDA network constructed by Sonawane et al. (2017). Network modules were identified using the top three algorithms from the Disease Module Identification, DREAM Challenge (Choobdar, et al., 2019): K1 (kernel clustering), M1 (modularity optimization), and R1 (random walk-based clustering). We implemented these algorithms through the MONET package (Tomasoni, et al., 2020) and applied each to the whole-blood-specific TF network maps from METANet and PANDA. The M1 algorithm was run in both directed and undirected modes by varying the "--linksdir" parameter. Summary characteristics of the modules generated by each algorithm and network are provided in Supplementary Table S6. The final modules used for the *SREBF2*-related biological analysis were derived from the directed M1 algorithm.

As input gene-level signals, we used transcriptome-wide association study (TWAS) p-values derived from the Long Life Family Study (LLFS). We considered 11 traits associated with cardiovascular risk, including body mass index (BMI), high-density lipoprotein (HDL), low-density lipoprotein (LDL), and triglycerides, among others. Replication was assessed using gene-level association statistics from the Framingham Heart Study (FHS) cohort (KANNEL, et al., 1979; Splansky, et al., 2007). Details on RNA-seq data processing and covariate adjustments have been previously described by Acharya et al. (Acharya, et al., 2025).

Across the 11 traits, FISHNET identified 114 FISHNET genes of which 24 were replicated. Using the whole blood tissue-specific PANDA network as input, we identified 0 FISHNET genes (see Supplementary Tables S7).

A central biological story emerging from these results centers on *SREBF2*, a replicated FISHNET gene negatively associated with HDL. Our findings support a model in which high HDL levels may suppress *SREBF2* activity, whereas low HDL permits *SREBF2* activation and sustains inflammatory signaling. *SREBF2* encodes SREBP2, a master transcription factor of sterol metabolism, canonically activated in adipocytes under low-cholesterol conditions (Bauer, et al., 2011; Horton, et al., 2002; Jiang, et al., 2005). Recent studies have shown that inflammatory stress can also activate SREBP2, enabling it to bind and induce inflammatory and interferon-response genes (Fowler, et al., 2023; Kusnadi, et al., 2019; Zhao, et al., 2011). In the whole blood tissue-specific METANet, *SREBF2* appears in the same module as other negatively HDL-associated FISHNET genes–*S100A9, S100A12, TLR8,* and *ITGB2*–all of which converge on pathways that activate the NADPH oxidase 2 (NOX2) complex. *S100A9* and *S100A12* are calgranulins, a type of alarmin, that can act as ligands for the receptor for advanced glycation end products (RAGE), triggering assembly and activation of the NOX2 complex (Bowman and Schmidt, 2011; Dong, et al., 2022; Xiao, et al., 2020). *TLR8* is a pattern recognition receptor that primes NOX2 activation in human neutrophils (Makni-Maalej, et al., 2015). *ITGB2* promotes leukocyte adhesion to the endothelium and triggers "outside-in" signaling that further promotes NOX2 assembly and activation (Chen, et al., 2016). Collectively, these genes link *SREBF2* to NOX2-based excessive production of reactive oxygen species (ROS), which oxidize HDL and convert it into a pro-inflammatory form (oxHDL) that contributes to atherosclerosis. Notably, *S100A9*, *S100A12*, and *TLR8* also activate NF-κB, inducing cytokines (TNFα and IL-1β) known to activate *SREBF2* (Cluzeau, et al., 2017; Qin, et al., 2006; Yang, et al., 2007; Zhang, et al., 2023). Together, these findings suggest a positive feedback loop in which reduced HDL allows *SREBF2* activation, which in turn promotes inflammatory cascades that further lower HDL, providing a mechanistic link between cholesterol metabolism, immune activation, and chronic low-grade inflammation characteristic of obesity.

REFERENCES

Acharya, S., *et al.* FISHNET: A Network-based Tool for Analyzing Gene-level P-values to Identify Significant Genes Missed by Standard Methods. *bioRxiv* 2025:2025.2001. 2029.635546.

Andersson, R., *et al.* An atlas of active enhancers across human cell types and tissues. *Nature* 2014;507(7493):455-461.

Bauer, S., *et al.* Sterol regulatory element-binding protein 2 (SREBP2) activation after excess triglyceride storage induces chemerin in hypertrophic adipocytes. *Endocrinology* 2011;152(1):26-35.

Bowman, M.A.H. and Schmidt, A.M. S100/calgranulins EN-RAGEing the blood vessels: implications for inflammatory responses and atherosclerosis. *American journal of cardiovascular disease* 2011;1(1):92.

Chen, S.-H., Oyarzabal, E.A. and Hong, J.-S. Critical role of the Mac1/NOX2 pathway in mediating reactive microgliosis-generated chronic neuroinflammation and progressive neurodegeneration. *Current opinion in pharmacology* 2016;26:54-60.

Choobdar, S., *et al.* Assessment of network module identification across complex diseases. *Nature methods* 2019;16(9):843-852.

Cluzeau, T., *et al.* Pro-inflammatory proteins S100A9 and tumor necrosis factor-α suppress erythropoietin elaboration in myelodysplastic syndromes. *haematologica* 2017;102(12):2015.

Dong, H., *et al.* Pathophysiology of RAGE in inflammatory diseases. *Frontiers in Immunology* 2022;13:931473.

Fowler, J.W.M., *et al.* SREBP2 regulates the endothelial response to cytokines via direct transcriptional activation of KLF6. *Journal of Lipid Research* 2023;64(8).

Horton, J.D., Goldstein, J.L. and Brown, M.S. SREBPs: activators of the complete program of cholesterol and fatty acid synthesis in the liver. *The Journal of clinical investigation* 2002;109(9):1125-1131.

Jiang, T., *et al.* Diet-induced obesity in C57BL/6J mice causes increased renal lipid accumulation and glomerulosclerosis via a sterol regulatory element-binding protein-1c-dependent pathway. *Journal of biological chemistry* 2005;280(37):32317-32325.

KANNEL, W.B., *et al.* AN INVESTIGATION OF CORONARY HEART DISEASE IN FAMILIES: THE FRAMINGHAM OFFSPRING STUDY. *American Journal of Epidemiology* 1979;110(3):281-290.

Kusnadi, A., *et al.* The cytokine TNF promotes transcription factor SREBP activity and binding to inflammatory genes to activate macrophages and limit tissue repair. *Immunity* 2019;51(2):241-257. e249.

Makni-Maalej, K., *et al.* TLR8, but not TLR7, induces the priming of the NADPH oxidase activation in human neutrophils. *Journal of Leucocyte Biology* 2015;97(6):1081-1087.

Qin, J., *et al.* TLR8-mediated NF-κB and JNK activation are TAK1-independent and MEKK3-dependent. *Journal of Biological Chemistry* 2006;281(30):21013-21021.

Sonawane, A.R., John Platig, Maud Fagny, Cho-Yi Chen, Joseph N Paulson, Camila M Lopes-Ramos, Dawn L DeMeo, John Quackenbush, Kimberly Glass, and Marieke Lydia Kuijjer. Gene regulatory networks for 38 human tissues. In. Zenodo; 2017.

Sonawane, A.R., *et al.* Understanding tissue-specific gene regulation. *Cell reports* 2017;21(4):1077-1088.

Splansky, G.L., *et al.* The third generation cohort of the National Heart, Lung, and Blood Institute's Framingham Heart Study: design, recruitment, and initial examination. *American journal of epidemiology* 2007;165(11):1328-1335.
The GTEx Consortium. The GTEx Consortium atlas of genetic regulatory effects across human tissues. *Science* 2020;369(6509):1318-1330.
Tomasoni, M., *et al.* MONET: a toolbox integrating top-performing methods for network modularization. *Bioinformatics* 2020;36(12):3920-3921.
Xiao, X., *et al.* S100 proteins in atherosclerosis. *Clinica chimica acta* 2020;502:293-304.
Yang, Z., *et al.* S100A12 provokes mast cell activation: a potential amplification pathway in asthma and innate immunity. *Journal of allergy and clinical immunology* 2007;119(1):106-114.
Zhang, X., *et al.* Proinflammatory S100A9 stimulates TLR4/NF-κB signaling pathways causing enhanced phagocytic capacity of microglial cells. *Immunology letters* 2023;255:54-61.
Zhao, L., *et al.* Inflammatory stress exacerbates hepatic cholesterol accumulation via increasing cholesterol uptake and de novo synthesis. *Journal of gastroenterology and hepatology* 2011;26(5):875-883.
